# Integrated Metabolomic and Genomic Analysis of the Antibacterial Mechanism of Postbiotics Derived from *Bacillus velezensis* 906 Against *Listeria monocytogenes*

**DOI:** 10.3390/foods15081364

**Published:** 2026-04-14

**Authors:** Zhe Liu, Xuetuan Wei, Qingyan Pan, Xifeng Zuo, Ping Chen, Ailing Guo

**Affiliations:** 1 College of Food Science and Technology, Huazhong Agriculture University, Wuhan 430070, China; 2 Suizhou Center for Disease Control & Prevention, Suizhou 441300, China

**Keywords:** *Bacillus velezensis*, antibacterial mechanism, anti-biofilm, cell membrane damage

## Abstract

Postbiotics derived from *Bacillus* species are recognized as promising natural antimicrobial agents. This study aimed to systematically evaluate the inhibitory activity of postbiotics derived from *B. velezensis* 906 against *L. monocytogenes*, elucidate the underlying antibacterial mechanisms using agar diffusion assays, broth microdilution, growth kinetics, flow cytometry, phospholipid competition assays, whole-genome mining, and non-targeted metabolomics, and characterize the bioactive metabolites responsible for their antibacterial effects. The postbiotics exhibited significant antagonistic activity against Gram-positive bacteria, Gram-negative bacteria, and fungi. They also inhibited pathogens such as *Salmonella* and *Enterobacter sakazakii*. Against *L. monocytogenes*, the minimum inhibitory concentration was 0.0083 mg/mL. At 1× MIC, the OD600 after 24 h remained at approximately 0.8, compared with 1.3–1.4 in the untreated control, whereas treatment at 4× MIC almost completely inhibited bacterial growth. Mechanistic analyses suggested that the postbiotics interact with membrane phospholipids, resulting in membrane disruption, increased intracellular reactive oxygen species accumulation, and enhanced membrane permeability. Integrated genome mining and non-targeted metabolomics indicated that the antibacterial activity was associated with a coordinated antimicrobial network involving lipopeptides, polyketides, bacteriocin-related compounds, and siderophore-associated metabolites. These findings provide insight into the antibacterial basis of *B. velezensis* 906 postbiotics and support their potential application in food safety control.

## 1. Introduction

In recent years, foodborne pathogen-associated contamination incidents have occurred frequently, posing serious threats to food safety and public health. Among these pathogens, *Listeria monocytogenes* is widely distributed in the natural environment and exhibits remarkable tolerance to various environmental stressors, including low pH, high osmotic pressure, and low temperatures. Consequently, it presents a relatively high risk of persistent transmission in frozen foods, lightly processed foods, and ready-to-eat foods with long shelf life. The World Health Organization has classified it as one of the four major foodborne pathogens [1]. This bacterium is the causative agent of listeriosis, a severe invasive disease that disproportionately affects pregnant women, neonates, the elderly, and immunocompromised individuals. Clinical manifestations include gastroenteritis, septicemia, meningitis, and miscarriage, with reported mortality rates ranging from 20–30% [2]. Furthermore, *L. monocytogenes* can form biofilms on food contact surfaces such as stainless steel, plastic, and rubber and can persist in food processing equipment, drainage systems, and other hard-to-clean niches for extended periods, thereby contributing to persistent contamination and increasing food safety risks [3]. Therefore, the development of safe and effective strategies to control *L. monocytogenes* contamination remains a critical priority for food safety management.

In recent years, postbiotics have attracted increasing attention as functional bioactive agents. They are generally defined as inactivated probiotic cells and their metabolic products, including cell-associated components, exopolysaccharides, short-chain fatty acids, antimicrobial peptides, and various organic acids [4]. Owing to the absence of viable microorganisms, postbiotics present several advantages, including high safety, excellent stability, and rapid onset of bioactivity. Notably, *Bacillus* spp. have emerged as an important microbial source for postbiotics due to their strong environmental adaptability, abundant secondary metabolism potential, and ability to produce diverse bioactive compounds. Kumar et al. reported that postbiotics derived from *B. amyloliquefaciens* COFCAU-P1 exhibited pronounced antibacterial and antioxidant activities [5]. These postbiotics not only inhibited a broad spectrum of pathogenic bacteria but also enhanced host defense by modulating immune-related responses. Meanwhile, *Bacillus pumilus* H2, isolated from marine sediments, was shown to produce extracellular postbiotic components with potent anti-*Vibrio* activity [6]. The active compound was identified as amicoumacin A, which exerts its antibacterial effect primarily by disrupting the integrity of the cell membrane. These findings underline the considerable potential of *Bacillus*-derived postbiotics in anti-infective applications and the development of natural antimicrobial agents.

Current research on *L. monocytogenes* primarily focuses on chemical and biological control strategies. Conventional chemical bactericides are widely applied due to their convenience and cost-effectiveness. However, their potential safety risks and residual effects remain concerns. In contrast, probiotics and their metabolites have emerged as promising biological protectants due to their multiple advantages, owing to their demonstrated safety, environmental compatibility, and broad-spectrum antibacterial activity [7]. *Bacillus* species, as common beneficial microorganisms, exhibit advantageous traits including heat resistance, acid tolerance, rapid growth, and high stability. Moreover, they produce diverse postbiotics with antibacterial properties, such as bacteriocins, lipopeptides, volatile compounds, and polyketides, making them a valuable source of natural antimicrobial agents [8]. Consequently, *Bacillus* species have been extensively applied in biological control, environmental protection, and healthcare [9]. These microorganisms demonstrate broad-spectrum inhibitory effects against various foodborne pathogens. Their antimicrobial metabolites disrupt bacterial membranes by compromising membrane integrity and permeability, leading to intracellular content leakage and eventual cell lysis. For example, the antibacterial peptide NDYT-8, isolated from the supernatant of *Bacillus subtilis* natto, increases membrane permeability and inhibits *Escherichia coli* and *Salmonella enteritidis* [10]. Similarly, postbiotics from *B. velezensis* BP1 enhance membrane permeability and induce metabolic imbalance, effectively suppressing *Staphylococcus aureus* and *Salmonella typhimurium* [1]. In addition, *B. coagulans* T242 exhibits broad-spectrum antibacterial activity, and its supernatant inhibits the growth of *Salmonella typhimurium* SL1344 and alters its cellular morphology [11].

Although previous studies have demonstrated that *Bacillus*-derived bioactive metabolites exert promising inhibitory effects against diverse pathogenic bacteria, research specifically targeting important foodborne pathogens such as *L. monocytogenes* remains relatively limited [12]. In particular, the compositional profiles of these bioactive metabolites, the accurate identification of their key functional components, and the synergistic antibacterial mechanisms among multiple constituents have not yet been systematically and comprehensively elucidated. In this context, the integrated application of genomics and metabolomics is highly warranted. Genomics enables the prediction of a strain’s secondary metabolic potential at the biosynthetic gene cluster level, laying a genetic foundation for the targeted mining of bioactive metabolites. Metabolomics allows systematic profiling of the overall metabolite composition and differential metabolites, providing direct evidence for identifying key active compounds in complex fermentation supernatants and clarifying their antibacterial mechanisms. Previous studies using *B. velezensis* FZB42 as a model strain have demonstrated that this strain possesses a wealth of biosynthetic gene clusters related to antimicrobial metabolites [13]. In addition, Wang et al. combined whole-genome sequencing with untargeted metabolomics to systematically explore the secondary metabolic potential of *B. velezensis* Q-426 and confirmed the actual production of multiple lipopeptide metabolites [12]. Collectively, these findings indicate that the integration of multi-omics approaches can effectively reveal the material basis and biosynthetic potential of bioactive metabolites from *Bacillus*, thus facilitating the identification and functional characterization of key antimicrobial components. Based on the above, in the study, a strain with strong antibacterial activity, identified as *B. velezensis* 906, was isolated from rice surfaces. Whole-genome sequence and non-targeted metabolomic analyses were employed to characterize the active components of its postbiotics and to elucidate their antimicrobial mechanisms. This study aimed to systematically evaluate the inhibitory effects of postbiotics derived from *B. velezensis* 906 against *L. monocytogenes* and to clarify the underlying mechanisms, thereby providing a theoretical basis for the development of probiotic-derived natural antimicrobial agents in food preservation.

## 2. Materials and Methods

### 2.1. Bacterial Strains and Culture Conditions

All microbial strains used in this study and their cultivation conditions are listed in Table S1. The experimental strains included *B. velezensis* 906, which was isolated from rice samples collected in Jiangxia District, Wuhan, Hubei Province, China; *B. cereus* ATCC 11778, *L. monocytogenes* ATCC 19114, *E. coli* ATCC 25922, and *S. aureus* ATCC 25923, which were obtained from the American Type Culture Collection (ATCC, Manassas, VA, USA); and *S. enteritidis*, *Salmonella muenchen*, *Colletotrichum acutatum*, *Colletotrichum gloeosporioides*, *Fusarium oxysporum*, and *E. sakazakii*, which were preserved in our laboratory collection. Luria–Bertani (LB), Tryptic Soy Broth (TSB), Potato Dextrose Agar (PDA) and Potato Dextrose Broth (PDB) media were purchased from LABLEAD Trading Co., Ltd. (Beijing, China).

### 2.2. Screening and Identification of B. velezensis 906

A total of ten rice samples were collected from Jiangxia District, Wuhan, Hubei Province, China (30°22′41.0″ N, 114°18′58.2″ E), in September. The samples were individually placed in sterile plastic bags, transported to the laboratory in a refrigerated container maintained at 4 °C, and subsequently stored at 4 °C until further use. All subsequent manipulations were performed under aseptic conditions. The samples were spread onto PDA plates and incubated at 28 °C for 96 h. During the cultivation of microorganisms from the rice surface, the isolation plates showed dense microbial growth with numerous colonies, which precluded reliable colony enumeration. Therefore, the primary screening was qualitative rather than quantitative and was based on the observation of visible inhibition halos against surrounding background microorganisms on the isolation plates. One isolate showing a pronounced inhibitory effect was selected, purified by repeated streaking on nutrient agar. Purified single colonies were inoculated into LB broth and cultured at 37 °C with shaking at 160 rpm for 24 h. Colony morphology was documented, and Gram staining was performed following standard microbiological protocols. Genomic DNA was extracted using a bacterial genomic DNA extraction kit (Tiangen Biotech, Beijing, China), the 16S rRNA gene was amplified using universal primers (27F/1492R), and the resulting amplicons were purified and sequenced by GenScript (Nanjing, China). Taxonomic identification was carried out by BLASTn (v2.17.0) searches against the National Center for Biotechnology Information nucleotide database, complemented with comparative analysis against type strains in EzTaxon-e (v2.1) [14]. Finally, a phylogenetic tree was constructed to determine the taxonomic classification of the strain.

### 2.3. Genomic Analysis of B. velezensis 906 and Antimicrobial Biosynthetic Gene Cluster Mining

Strain 906 was sequenced using Oxford Nanopore technology [15]. Genomic DNA was extracted with a Qiagen DNA extraction kit (Qiagen, Hilden, Germany) according to the manufacturer’s protocol. DNA integrity and purity were evaluated by 0.75% agarose gel electrophoresis, a NanoDrop spectrophotometer (Thermo Fisher Scientific, Waltham, MA, USA), and a Qubit fluorometer (Thermo Fisher Scientific, Waltham, MA, USA). A sequencing library was subsequently prepared using a DNA library preparation kit, and the library concentration was quantified with a Qubit fluorometer. The final library was diluted to the appropriate concentration, loaded onto a flow cell, and sequenced in real time on the PromethION platform to generate raw sequencing data.

Protein-coding sequences were annotated by BLASTp (v2.17.0) against the Kyoto Encyclopedia of Genes and Genomes (KEGG), Reference Sequence Database (RefSeq), and Clusters of Orthologous Groups (COGs) databases. In addition, genome sequencing coverage, GC content, GC skew, and overall genomic architecture were analyzed and visualized using Circos software (0.69-10). Putative biosynthetic gene clusters associated with antimicrobial metabolites were predicted using the antiSMASH online platform (https://antismash.secondarymetabolites.org/#!/start (accessed on 10 February 2026)).

### 2.4. Preparation and Antimicrobial Spectrum of Postbiotics from B. velezensis 906

The postbiotics were prepared following the protocol described by Arrioja-Bretón et al. (2020) with minor modifications [16]. Briefly, a single colony of *B. velezensis* 906 was inoculated into 20 mL of LB broth and incubated at 37 °C with shaking at 180 rpm for 12 h. A 1% (*v*/*v*) inoculum from this culture was subsequently transferred into 100 mL fresh LB medium (initial pH 7.0) in a 250 mL Erlenmeyer flask and incubated under the same conditions for an additional 28 h to ensure adequate bacterial growth and metabolite production. After fermentation, the culture broth (final pH 7.0) was centrifuged at 10,000 rpm for 20 min at 4 °C. The collected supernatant was sterile-filtered through a 0.22 µm membrane to obtain the postbiotics. The filtrate was then lyophilized, and the resulting powder was weighed and reconstituted in sterile water to a final concentration of 0.134 mg/mL. The working solution was stored at −80 °C until further use.

The antimicrobial spectrum of the postbiotics was evaluated according to the method described by Barreiros et al. (2022) with minor modifications [17]. Representative microorganisms included Gram-positive bacteria (*L. monocytogenes*, *S. aureus*, and *B. cereus*), Gram-negative bacteria (*E. sakazakii*, *E. coli*, and *S. enteritidis*), and fungi (*F. oxysporum*, *C. gloeosporioides*, and *C. acutatum*). Each strain was cultured in the appropriate growth medium at its optimal temperatures until reaching the logarithmic phase. Antimicrobial activity was then determined using the agar diffusion assay.

Briefly, the test microorganisms, including Gram-positive bacteria, Gram-negative bacteria, and fungi, were cultured in the appropriate media under their optimal conditions until the logarithmic phase. The indicator suspensions were standardized to 1 × 10^6^ CFU/mL. Then, 1.5 mL of each standardized suspension was mixed with 13.5 mL of molten agar medium and poured into sterile Petri dishes to prepare indicator lawns. After solidification, sterile Oxford cups (6 mm in diameter) were placed on the agar surface, and 150 μL of postbiotics was added to each cup. After incubation under the appropriate conditions, the diameters of the inhibition zones were measured in millimeters. Antimicrobial activity was evaluated based on the size of the inhibition zone.

### 2.5. Determination of the Antibacterial Activity of Crude Extracts

Antibacterial substances were extracted from the postbiotics by ammonium sulfate precipitation. Briefly, 30 mL of the supernatant was gradually supplemented with solid ammonium sulfate to final saturation levels of 30%, 40%, 50%, 60%, and 70%. The mixtures were incubated at 4 °C for 12 h and then centrifuged at 12,000 rpm for 20 min at 4 °C. The resulting precipitates were reconstituted in phosphate-buffered saline (PBS) to obtain the crude antimicrobial peptide extracts.

The antibacterial activity of the crude extracts was evaluated using the Oxford cup diffusion assay [18]. Specifically, *L. monocytogenes* cultures were standardized to 1 × 10^6^ CFU/mL. A sterile Oxford cup (6 mm in diameter) was placed vertically at the center of a sterile Petri dish. Subsequently, 1.5 mL of the standardized bacterial suspension was mixed with 13.5 mL of molten TSB agar and poured into the dish to form a uniform bacterial lawn. After solidification, the Oxford cup was carefully removed to generate a standardized well. Then, 150 μL of each test sample was added to the well. The plates were incubated at 37 °C for 24 h, and the diameters of the inhibition zones (mm) were measured to assess antibacterial activity.

### 2.6. Antibacterial Activity of Crude Extracts

To comprehensively evaluate the antibacterial activity of the crude extracts derived from *B. velezensis* 906, six representative indicator microorganisms were selected, including *L. monocytogenes*, *S. aureus*, *S. muenchen*, *E. coli*, *Mucor* spp., and *Penicillium* spp. The antibacterial activities of the crude extracts and the corresponding postbiotics were determined using the Oxford cup diffusion assay.

### 2.7. Study on the Potential Mechanism of Postbiotics Against L. monocytogenes

#### 2.7.1. Minimum Inhibitory Concentration

The antibacterial activity of the postbiotics against *L. monocytogenes* was quantitatively evaluated using the broth microdilution method in accordance with the guidelines of the Clinical and Laboratory Standards Institute (CLSI) [19]. Briefly, two-fold serial dilutions of the postbiotic were prepared in 96-well microtiter plates to obtain final concentrations ranging from 132 μg/mL to 0.016 μg/mL. Each well contained an equal volume of postbiotic solution and bacterial suspension (1 × 10^6^ CFU/mL). The plates were incubated at 37 °C for 24 h, and bacterial growth was assessed by measuring the optical density at 600 nm (OD_600_). TSB without inoculation served as the blank control, while bacterial suspension without postbiotics served as the growth control. Bacterial growth was first assessed visually based on broth turbidity and then confirmed spectrophotometrically by measuring OD_600_ using a microplate reader (Thermo Scientific, Singapore). The MIC was defined as the lowest concentration at which the broth remained clear, showing no visible bacterial growth or significant increase in OD_600_. The measurements were performed in triplicate.

#### 2.7.2. Effect of the Postbiotics on the Growth Kinetics of *L. monocytogenes*

To evaluate the effects of postbiotics on bacterial growth kinetics, an overnight culture of *L. monocytogenes* was diluted in sterile TSB to an OD_600_ of 0.6–0.8. Three experimental groups were established: a blank control group (80 μL bacterial suspension + 80 μL PBS), a 1× MIC treatment group (80 μL bacterial suspension + 80 μL 2× MIC postbiotic solution), and a 2× MIC treatment group (80 μL bacterial suspension + 80 μL 4× MIC postbiotic solution). Samples were incubated at 37 °C with shaking at 60 rpm. The OD_600_ was recorded every 2 h using a microplate reader to generate growth curves.

#### 2.7.3. Effect of *B. velezensis* Postbiotics on Cell Integrity of *L. monocytogenes*

1. Alkaline phosphatase (AKP) activity

AKP activity was determined to evaluate cell wall integrity following postbiotic treatment. Overnight cultures were centrifuged at 3000 rpm for 15 min, washed with PBS and resuspended to an initial OD_600_ of 0.6–0.8. The experimental groups included a blank control and treatment groups at final concentrations of 1× MIC and 2× MIC. All samples were incubated at 37 °C for 370 min, with aliquots collected every 30 min. Each aliquot was centrifuged at 8000 rpm for 15 min to obtain the supernatant, and AKP activity was determined according to the instructions provided with the AKP assay kit (Nanjing Jiancheng Bioengineering Institute, Nanjing, China).

2. Leakage of intracellular nucleic acids and proteins

Membrane permeability was evaluated by measuring the release of intracellular nucleic acids and proteins as previously described [20]. Log-phase cells were harvested, washed three times with sterile PBS, and resuspended to an OD_600_ of 0.6–0.8. Postbiotics were added to achieve final concentrations of 0× MIC, 0.5× MIC, and 2× MIC, with PBS-treated cells serving as the negative control. Samples were incubated at 37 °C with shaking for 4 h. The absorbance of the supernatants was measured at 260 nm and 280 nm at 30 min intervals to quantify nucleic acid and protein leakage, respectively.

3. Cytoplasmic membrane depolarization

The effect of the postbiotics on the membrane depolarization of *L. monocytogenes* was investigated using the fluorescent probe DiSC_3_(5) (Meilun Biotech, Dalian, China). *L. monocytogenes* was cultured at 37 °C with shaking at 180 rpm for 8 h. Subsequently, aliquots (150 μL) of the bacterial suspension (1 × 10^8^ CFU/mL) were transferred into black 96-well plates, followed by the addition of 50 μL DiSC_3_(5) solution (10 μM). After incubation at 37 °C for 30 min to allow dye incorporation into the cytoplasmic membrane, postbiotics were added to final concentrations of 0.5× MIC, 1× MIC, 2× MIC, and 4× MIC. Fluorescence intensity was monitored at 1 min intervals. The excitation and emission wavelengths were set at 622 nm (slit width 10 nm) and 670 nm (slit width 5 nm), respectively [21].

#### 2.7.4. Effects of *B. velezensis* 906 Postbiotics on the Integrity of *L. monocytogenes* Cell Membranes

1. Flow cytometry analysis

Cell membrane integrity was evaluated by flow cytometry using propidium iodide (PI) staining, as described by Shahryari et al. (2018) [22]. *L. monocytogenes* cultures were diluted in sterile PBS to an OD_600_ of 0.6–0.8 and treated with postbiotics at final concentrations of 3 × MIC and 5 × MIC. Cells treated with PBS alone served as the negative control. After incubation at 37 °C for 12 h, cells were harvested by centrifugation, washed with PBS and resuspended in PBS. The cells were then incubated with PI solution (50 µg/mL) for 30 min in the dark at room temperature, followed by centrifugation at 3000 rpm for 3 min, washing three times with PBS, and resuspension in buffer. Finally, cell membrane permeability and integrity were analyzed using a CytoFLEX flow cytometer (Beckman Coulter, Beijing, China).

2. Morphological analysis by SEM and AFM [23]

To investigate the effects of the fermentation supernatant on the cell morphology of *L. monocytogenes*, scanning electron microscopy (SEM) (Hitachi High-Tech, Tokyo, Japan) and atomic force microscopy (AFM) (Bruker, Billerica, MA, USA) were employed for characterization. Briefly, an overnight-cultured *L. monocytogenes* suspension was diluted to 1 × 10^6^ CFU/mL, followed by the addition of postbiotics to achieve a final concentration of 4× MIC. The samples were incubated at 37 °C for 2 h and 10 h respectively, with untreated bacterial suspensions serving as controls. After treatment, the samples were fixed with 2.5% glutaraldehyde at 4 °C for 12 h, dehydrated using graded ethanol solutions, dried, and gold-coated before SEM observation to analyze changes in cell surface morphology. Sample preparation for AFM analysis followed the same fixation and dehydration protocol. Imaging was conducted using a Bruker (Multimode 8, Barbara, CA, USA) AFM with PeakForce Tapping mode. The probe cantilever had a resonant frequency range of 45–95 kHz and an elastic constant of 0.2–0.8 N/m. All acquired images and data were processed and analyzed using NanoScope Analysis software. (v1.5)

#### 2.7.5. Detection of Intracellular Reactive Oxygen Species (ROS)

Intracellular ROS levels in *L. monocytogenes* were determined using the fluorescent probe 2′,7′-dichlorofluorescin diacetate (DCFH-DA) [24]. *L. monocytogenes* was cultured overnight at 37 °C with shaking at 160 rpm, harvested, washed, and resuspended in PBS (0.01 M, pH 7.4). The bacterial suspension was mixed with an equal volume of DCFH-DA solution (10.0 μM) and incubated at 37 °C for 30 min in the dark to facilitate probe penetration into the cells. After staining, the cells were washed three times with PBS to remove unbound dye. The labeled cells were then mixed with postbiotics solutions at final concentrations of 0.5× MIC, 1× MIC, 2× MIC, and 4× MIC and incubated for 30 min. ROS levels was quantified by fluorescence intensity at an excitation wavelength of 488 nm and an emission wavelength of 525 nm.

#### 2.7.6. Inhibitory Effects of Lipids or Peptidoglycan (PGN) on Antibacterial Activity

To further elucidate the membrane-targeting mechanism of the postbiotics, the potential antagonistic effects of exogenously added membrane phospholipids and cell wall components were evaluated [25]. The postbiotics were preincubated with varying concentrations of phosphatidylglycerol (PG), phosphatidylethanolamine (PE), or PGN and incubated at 37 °C for 60 min. Subsequently, 100 μL of each mixture was transferred into 96-well microplates, followed by the addition of an equal volume of *L. monocytogenes* suspension (1.0 × 10^6^ CFU/mL). The plates were incubated statically at 37 °C for 16–18 h, and the MIC of the metabolites under different treatments was determined to evaluate the potential antagonistic effects of lipids or PGN on their antibacterial activity. In addition, the postbiotics were incubated with PG and PE at a final concentration of 64 μg/mL at 37 °C for 60 min to further confirm their targets. The mixtures were then applied to Oxford cups on agar plates, and inhibition zone diameters were recorded after overnight incubation at 37 °C to evaluate the effect of phospholipid binding on antibacterial activity.

#### 2.7.7. Effects of *B. velezensis* Postbiotics on Biofilm Formation by *L. monocytogenes*

The inhibitory effect of the postbiotics on biofilm formation was evaluated [26,27]. Overnight cultures of *L. monocytogenes* were inoculated into TSB and transferred into 12-well plates containing sterile glass coverslips. The experimental groups were treated with postbiotics at final concentrations of 1/4× MIC, 1/2× MIC, and 4× MIC, while untreated cultures served as the control. All samples were incubated statically at 37 °C for 24 h, and biofilm formation was assessed using the crystal violet staining method. Briefly, the coverslips were carefully removed and washed three times with sterile PBS to eliminate non-adherent cells. The attached biofilms formed on the glass surfaces were then stained with 0.1% (*w*/*v*) crystal violet solution for 20 min, rinsed, and air-dried. Biofilm morphology was observed under a light microscope. Subsequently, the bound crystal violet was solubilized in 95% ethanol, and absorbance was measured at 570 nm using a microplate reader (Multiskan SkyHigh, Shanghai, China). The biofilm inhibition was calculated according to the following equation:

(1) $$\mathrm{Inhibition}\;\mathrm{rate}\;(\%)\;=\;\frac{ODcontrol-ODtreated}{ODcontrol}\times 100$$

### 2.8. Metabolomics Analysis

#### 2.8.1. Metabolomic Profiling of the Postbiotics from *B. velezensis* 906

Non-targeted metabolomic analysis was conducted to characterize the secreted metabolites contributing to the antagonistic activity of the postbiotics, following the method described by Zha et al. (2024) [28], with minor modifications. Briefly, lyophilized postbiotics samples were reconstituted, and 100 μL aliquots were mixed with 400 μL methanol– acetonitrile (3:1, *v*/*v*). The mixtures were vortexed for 5 min, sonicated for 15 min, and incubated at 4 °C for 1 h to ensure complete metabolite extraction. Subsequently, the samples were centrifuged at 12,000 rpm (4 °C) for 15 min, and 100 μL of the supernatant was collected and evaporated to dryness under vacuum. The residues were reconstituted in 50 μL of methanol–water solution (1:1, *v*/*v*), vortexed for 3 min, and centrifuged. The resulting supernatants were analyzed using liquid chromatography–mass spectrometry (LC-MS).

The chromatographic separation was performed on a Waters HSS T3 column (100 × 2.1 mm, 1.8 μm) under the following conditions: The mobile phase consisted of (A) ultrapure water containing 0.1% formic acid and (B) acetonitrile containing 0.1% formic acid. The flow rate was set at 0.3 mL/min, the column temperature was maintained at 40 °C, and the injection volume was 2 µL. The gradient elution program was as follows: 0–1 min, 100% A; 1–12 min, linear decrease to 5% A (95% B); 12–13 min, hold at 5% A; 13.0–13.1 min, return to 100% A; and 13.1–17 min, re-equilibration at 100% A. All samples were kept at 4 °C in the autosampler throughout the analysis. Samples were analyzed in a randomized sequence, and quality control samples were interspersed at regular intervals to monitor system stability and ensure data reliability. The Q Exactive mass spectrometer (Thermo Fisher Scientific, Waltham, MA, USA) was operated in ESI+/ESI− mode with sheath gas flow rate of 30 arb, aux gas flow rate of 10 arb, capillary temperature of 325 °C, spray voltage of 3.50 kV and −2.50 kV for ESI+ and ESI−, respectively.

#### 2.8.2. Bioinformatics Analysis

Raw LC-MS data were imported into Progenesis QI software 2.0 (Waters Co., Ltd., Milford, MA, USA) for peak alignment, normalization, and metabolite identification. Metabolites were annotated by matching accurate mass and fragmentation patterns against the Human Metabolome Database (HMDB) (http://www.hmdb.ca/) (accessed on 10 February 2026) and the KEGG database (http://www.genome.jp/kegg/) (accessed on 10 February 2026). The processed data matrix was uploaded to the Tutu cloud platform (https://cloudtutu.com) (accessed on 10 February 2026) for multivariate statistical and bioinformatics analyses. Principal component analysis (PCA) was performed to evaluate the overall metabolic differences between groups. Differential metabolites were screened based on variable importance in projection (VIP) > 1, fold change (FC) ≥ 3 or ≤1/3, and *p* < 0.05. Volcano plot analysis, Z-score analysis, and KEGG pathway enrichment analysis were subsequently performed to characterize the differential metabolites and identify the metabolic pathways associated with them. Because no authentic standards were analyzed under identical experimental conditions, the reported compounds should be regarded as putatively annotated metabolites rather than unequivocally identified metabolites.

### 2.9. Statistical Analysis

All experiments were conducted in triplicate unless otherwise stated. Data are presented as the mean ± standard deviation (SD). Statistical comparisons among groups were evaluated using one-way analysis of variance (ANOVA) followed by Tukey’s post hoc test in SPSS software 26 (IBM Corp., Armonk, NY, USA). A *p*-value < 0.05 was considered statistically significant.

## 3. Results

### 3.1. Isolation and Identification of B. velezensis 906

**Figure 1:**
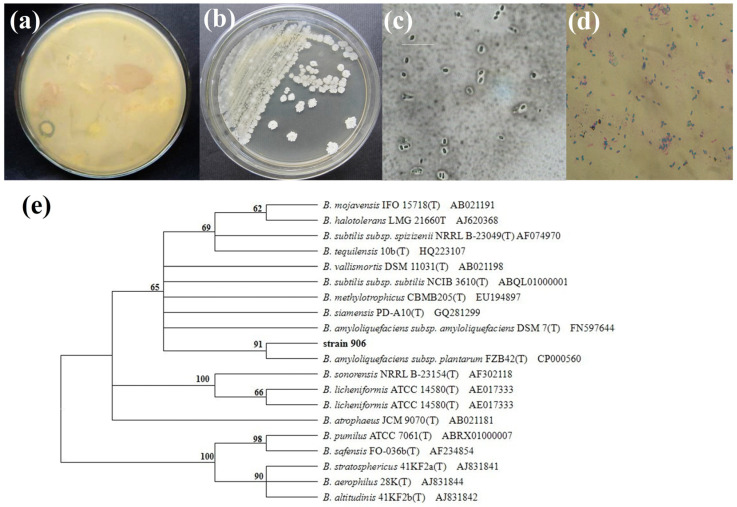
Discovery of strain (**a**), colony morphology (**b**), capsule stain morphology (**c**), spore stain morphology (**d**), and phylogenetic tree (**e**) of *B. velezensis* 906. Bootstrap values (%) are shown at the nodes.

During the cultivation of microorganisms from rice surface samples, one unknown colony exhibiting a clear inhibitory halo against surrounding background microorganisms on the isolation plate was observed (Figure 1a). The strain formed nearly circular colonies with wrinkled, white, and opaque surfaces. Microscopic examination indicated that the cells were Gram-positive, short rod-shaped, and capable of forming spores and capsules (Figure 1b–d). The 16S rRNA gene sequence of strain 906 was analyzed using the EzBioCloud database, and a phylogenetic tree was constructed based on sequence similarity (Figure 1e). Sequence alignment demonstrated that the 16S rRNA of strain 906 shared 99.9% sequence identity with *B. velezensis* FZB42 (GenBank accession number CP000560). The amplified 16S rRNA sequence was 1512 bp in length, and the sequence of strain 906 has been deposited in GenBank under accession number JN566073. To further support its taxonomic assignment, whole-genome comparative analysis was performed using representative genomes of closely related *Bacillus* species. Strain 906 possessed a genome of 3,954,104 bp with a GC content of 46.64%. Genome-level k-mer comparison using Mash indicated that strain 906 was most closely related to *B. velezensis* FZB42, with a Jaccard similarity of 0.5438 and a Mash distance of 0.0234, corresponding to an estimated average nucleotide identity (ANI) of approximately 97.66%, which is clearly above the 95% threshold for species assignment. Therefore, strain 906 was identified as *B. velezensis* 906.

### 3.2. Genome Sequencing of B. velezensis 906 and Analysis of Antimicrobial Biosynthetic Gene Clusters

#### 3.2.1. Genome Sequencing and Assembly

**Figure 2:**
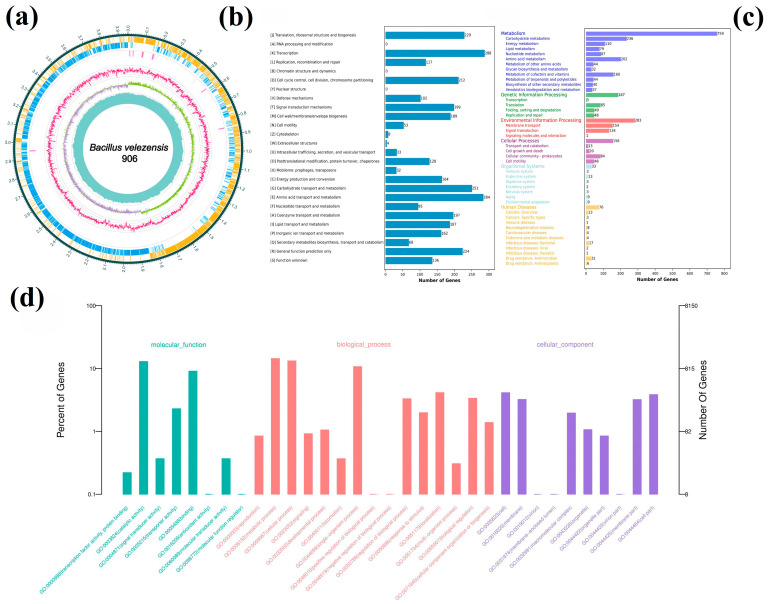
(**a**) Circular genome map of strain 906. From outside to inside, the circles represent coding sequences on the forward strand (yellow), coding sequences on the reverse strand (blue), tRNA genes (orange), rRNA genes (purple), CRISPR loci (light blue), genomic islands (green), GC content (magenta), GC skew (green for positive values and purple for negative values), and sequencing depth (cyan). (**b**) COG function classification statistics of genome coding protein of *B. velezensis* 906. (**c**) KEGG function classification statistics of genome coding protein of *B. velezensis* 906. (**d**) GO function classification statistics of genome coding protein of *B. velezensis* 906.

Whole-genome sequencing of *B. velezensis* 906 was performed using a single Flow Cell on the Oxford Nanopore PromethION platform. A total of 2,538,395,694 bp of raw sequencing data were generated, of which 2,173,478,037 bp were retained after quality filtering. De novo genome assembly followed by polishing and error correction yielded a high-quality complete genome. The assembled genome consists of a single circular chromosome of 3,954,104 bp with a GC content of 46.64% (Figure 2a and Table S2).

#### 3.2.2. Genome Functional Annotation

Genome prediction revealed that there were 3809 genes of coding proteins, accounting for 88.62% of the complete genome. Functional annotation of the predicted genes was performed using the COG, KEGG, GO, RefSeq, Pfam, and TIGRFAMs databases. A total of 2988 genes were assigned to 23 COG functional categories, accounting for 78.45% of the coding sequences (Figure 2b). According to the COG annotation, more genes were involved in transcription (category K, 288 genes) and amino acid transport and metabolism (category E, 284 genes), followed by carbohydrate transport and metabolism (category G, 251 genes) and genes responsible for translation, ribosomal structure and biosynthesis (category J, 229 genes). The COG annotation provides the basis for the synthesis, modification and transportation of lipopeptides and polypeptide secondary metabolites.

In the KEGG database, 2219 genes (58.26%) were divided into six categories (Figure 2c). Genes involved in metabolism constituted the largest proportion (34.20%), including 202 genes (9.10%) associated with amino acid metabolism. This finding is consistent with the COG annotation and reflects the strong metabolic potential of strain 906 for the biosynthesis of secondary metabolites. A total of 2278 coding proteins were annotated by the GO database, which were divided into three categories: molecular function, biological process and cellar component (Figure 2d). The predominance of genes related to catalytic activity, binding, metabolic processes, and cellular processes reflects the active metabolic profile and growth characteristics of *B. velezensis* 906.

#### 3.2.3. AntiSMASH Analysis

**Figure 3:**
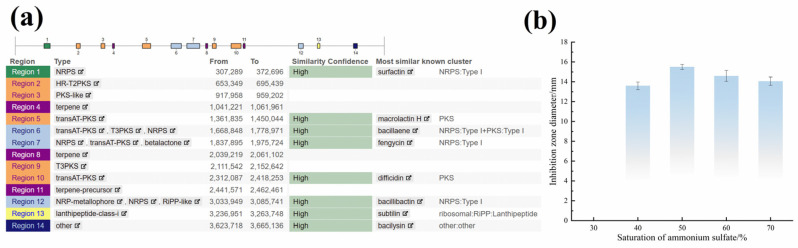
(**a**) Secondary metabolic potential of *B. velezensis* 906. (**b**) Inhibition zone diameters of antibacterial extracts obtained at different ammonium sulfate saturation levels of the prebiotics.

Based on systematic genome mining of the complete genome of *B. velezensis* 906 using antiSMASH, a total of 14 secondary metabolite biosynthetic gene clusters (BGCs) were identified (Figure 3a), which are widely distributed across the chromosome. The predicted product types include nonribosomal peptide synthetases (NRPSs), polyketide synthases (PKSs, including trans-AT PKS, type III PKS, and type II PKS-like clusters), terpenes, RiPP/lanthipeptides, metallophores/NRP-metallophores, and other biosynthetic categories, indicating substantial secondary metabolic diversity in this strain. Comparison with the minimum information about a biosynthetic gene cluster (MIBiG) database revealed that eight of these BGCs exhibited high similarity to previously characterized gene clusters, including surfactin (region 1, ~82%), macrolactin (region 5, ~100%), bacillaene (region 6, ~100%), fengycin (region 7, ~100%), difficidin (region 10, ~100%), bacillibactin (region 12, ~100%), subtilin (region 13, ~100%), and bacilysin (region 14, ~100%). The presence of these conserved gene clusters suggests that strain 906 retains the canonical capacity of *Bacillus* species to synthesize antimicrobial lipopeptides, polyketides, and small-molecule antibiotics, thereby providing a genomic basis for its broad-spectrum antibacterial phenotype.

In addition to the conserved lipopeptide and bacteriocin clusters, genome mining identified several orphan type II/type III PKS and terpene-associated BGCs lacking close MIBIG matches. Type II PKS systems are typically associated with aromatic polyketide biosynthesis, whose products frequently interfere with redox homeostasis, DNA-associated processes, and electron transport. Such mechanisms may potentiate oxidative stress responses and promote intracellular ROS accumulation under antimicrobial exposure. Type III PKS systems, characterized by single-enzyme iterative condensation reactions, often generate small aromatic metabolites with regulatory or quorum-sensing–interfering properties, potentially contributing to biofilm suppression rather than direct bactericidal activity. Meanwhile, terpene synthase-containing clusters may encode hydrophobic isoprenoid-like derivatives with the potential to influence membrane properties, such as fluidity and permeability. Therefore, these orphan BGCs may be associated with postbiotics-induced membrane damage, ROS accumulation, and biofilm inhibition. However, this potential relationship remains tentative and requires further validation. Overall, these orphan BGCs may indicate additional biosynthetic potential in strain 906, but their precise contribution to the antibacterial phenotype remains to be clarified by further targeted experiments.

### 3.3. Extraction and Antibacterial Evaluation of Postbiotics from B. velezensis 906

The effect of different ammonium sulfate saturation levels on the inhibitory activity of crude extracts against *L. monocytogenes* was assessed. The results showed that extracts obtained at 40–70% ammonium sulfate saturation exhibited different inhibitory effects. The extract prepared at 50% saturation displayed the strongest inhibitory activity, as indicated by the largest inhibition zone diameter (15.8 ± 0.46 mm), which was significantly higher than those observed for the 40%, 60%, and 70% saturation groups (*p* < 0.05) (Figure 3b). This result indicates that ammonium sulfate at 50% saturation was optimal for precipitating bioactive antimicrobial components. These findings establish a methodological basis for ammonium sulfate–mediated extraction and support subsequent process optimization.

**Figure 4:**
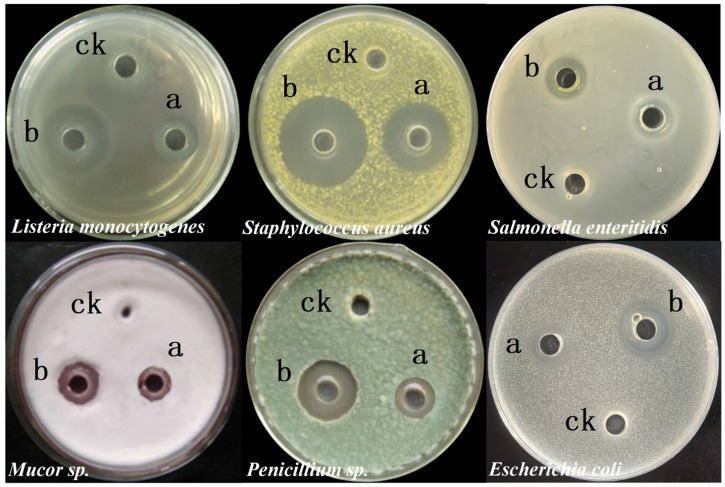
Antibacterial activity of the postbiotics and crude extract from *B. velezensis* 906 (a: postbiotics; b: crude extract; ck: blank control).

In addition, *B. velezensis* 906 exhibited broad-spectrum antagonistic activity (Figure 4). Both the postbiotics and the crude extract inhibited Gram-positive bacteria (*L. monocytogenes*, *S. aureus*), Gram-negative bacteria (*S. muenchen*, *E. coli*), and fungi (*Mucor* spp., *Penicillium* spp.). The postbiotics generated inhibition zones ranging from 10.53 ± 0.55 to 23.70 ± 0.46 mm, whereas the crude extract exhibited overall stronger antibacterial activity, including inhibition zones of 25.14 ± 1.00 mm against *L. monocytogenes* and 30.87 ± 0.83 mm against *Staphylococcus* sp. Therefore, inhibition zones produced by the crude extract were generally larger than those of the postbiotics, suggesting that ammonium sulfate precipitation effectively concentrated active antibacterial components. The inhibitory effect was stronger against Gram-positive bacteria than Gram-negative bacteria, which may be attributed to differences in cell envelope structure, particularly the absence of an outer membrane in Gram-positive organisms. Furthermore, the observed antifungal activity further indicates that the secreted metabolites likely include proteinaceous or lipopeptide antimicrobial compounds with broad-spectrum activity. Collectively, these results provide a foundation for further purification and structural characterization of the active components and highlight the potential application of *B. velezensis* 906 in food preservation and biological control.

### 3.4. Antimicrobial Spectrum of the Postbiotics

**Figure 5:**
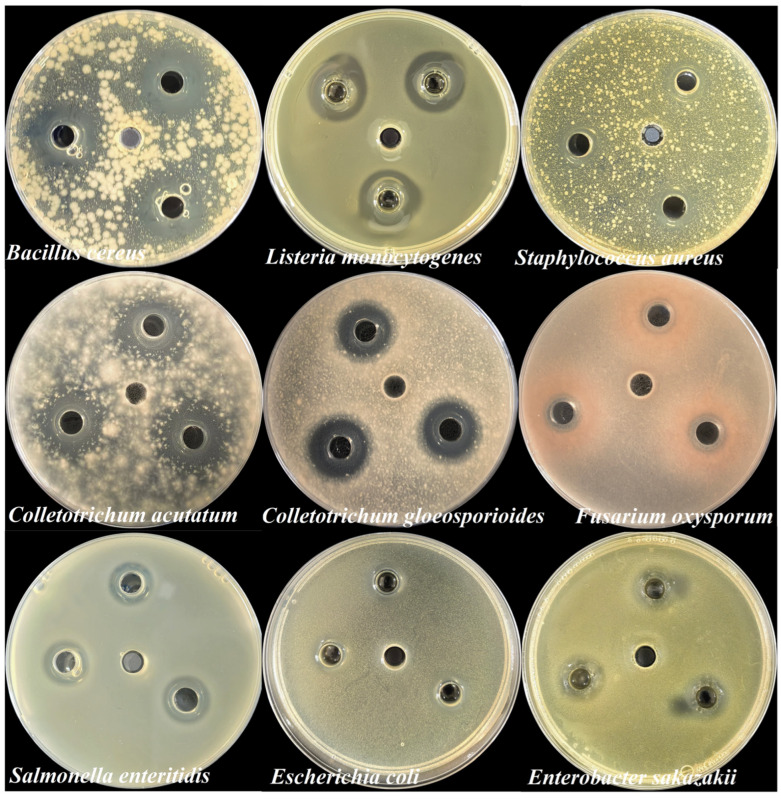
Antibacterial activity of postbiotics against *B. cereus*, *L. monocytogenes*, *S. aureus*, *C. acutatum*, *C. gloeosporioides*, *F. oxysporum*, *S. enteritidis*, *E. coli* and *E. sakazakii*.

Antimicrobial assays revealed that the postbiotics derived from *B. velezensis* 906 exhibited broad-spectrum inhibitory activity against diverse pathogenic microorganisms (Figure 5). Although many *Bacillus*-derived metabolites primarily inhibit Gram-positive bacteria, the postbiotics from strain 906 demonstrated significant activity against representative Gram-negative bacteria, including *S. enteritidis*, *E. coli*, and *E. sakazakii.* In addition, the postbiotics exhibited pronounced antagonistic effects against primary fruit-associated phytopathogenic fungi, including those affecting mango and pitaya, as well as *F. oxysporum* from banana. Inhibition zone assays confirmed that the postbiotics effectively restricted hyphal extension in these fungi, highlighting its broad antifungal capacity. Notably, these phytopathogens are responsible for severe postharvest diseases, including soft rot and browning, which result in substantial economic losses for the fruit industry. Therefore, the metabolites identified in this study represent promising candidates for environmentally friendly postharvest disease management. In summary, the postbiotics of *B. velezensis* 906 overcome the narrow antibacterial spectrum typically associated with many *Bacillus* species and exhibit considerable potential for applications in food safety and fruit preservation.

### 3.5. Analysis of the Potential Mechanism of Postbiotics Inhibiting L. monocytogenes

#### 3.5.1. Antibacterial Activity of the Postbiotics Against *L. monocytogenes*

**Figure 6:**
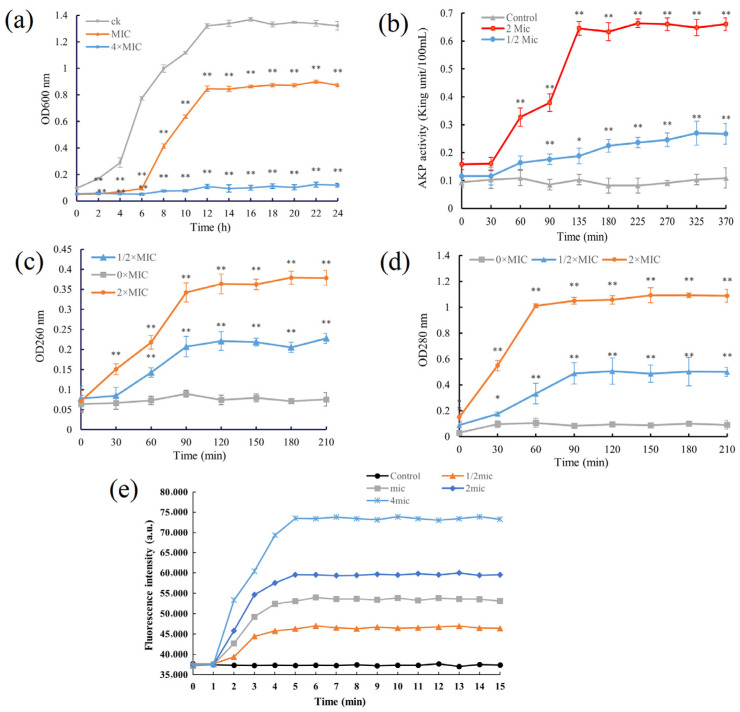
(**a**) The growth kinetics curve of the postbiotics at different concentrations on *L. monocytogenes*. (**b**) Changes in AKP activity in *L. monocytogenes* treated with postbiotics. (**c**) Effect of postbiotics on the leakage of intracellular nucleic acid of *L. monocytogenes*. (**d**) Effect of postbiotics on the leakage of intracellular protein of *L. monocytogenes*. (**e**) Assessment of cytoplasmic membrane depolarization analysis induced by postbiotics using DiSC_3_(5). Error bars represent the standard deviation of three independent replicates (*, *p* < 0.05; **, *p* < 0.01).

The MIC of the postbiotics against *L. monocytogenes* was determined to be 0.0083 mg/mL. Bacterial growth kinetics at different concentrations were monitored (Figure 6a). In the untreated control group, *L. monocytogenes* exhibited a typical growth profile, with OD600 values increasing rapidly between 4 and 12 h, entering the exponential phase, and stabilizing at approximately 1.3–1.4 after 24 h. In contrast, treatment with 1× MIC markedly delayed bacterial proliferation, showing minimal growth during the first 6 h. Growth subsequently entered a prolonged and attenuated exponential phase, ultimately reaching a stable OD600 of approximately 0.8, which was substantially lower than that of the control group. These results indicated that treatment at 1× MIC primarily exerted a bacteriostatic effect. At a higher concentration (4× MIC), bacterial growth was almost completely inhibited, with OD600 values remaining near baseline (<0.2) throughout the incubation period, demonstrating high bactericidal activity. Collectively, these results show that the postbiotics of *B. velezensis* 906 exert concentration-dependent inhibitory effects against *L. monocytogenes*, with higher concentrations effectively suppressing bacterial proliferation and inducing bactericidal activity.

#### 3.5.2. Effect of Postbiotics Metabolites on the Integrity of *L. monocytogenes* Cells

1. Determination of AKP activity

AKP is an enzyme primarily located in the periplasmic space between the cell wall and cytoplasmic membrane. When cell wall integrity is compromised, AKP is released into the extracellular environment, leading to increased detectable enzyme activity. Therefore, AKP activity serves as an indicator of bacterial structural integrity [29]. The results demonstrated that treatment with the postbiotics of *B. velezensis* 906 damaged the cellular structure of *L. monocytogenes* in a concentration-dependent manner. In the control group, AKP activity remained consistently low, indicating intact cell membranes with no significant disruption. In contrast, treatment with increasing concentrations of the postbiotics caused a progressive rise in AKP activity, which stabilized at significantly higher levels, indicating severe structural damage and substantial leakage of intracellular enzymes (Figure 6b). These findings suggest that the antibacterial activity of the *B. velezensis* 906 postbiotics is closely associated with impairment of cell wall integrity and increased membrane permeability. This mechanism is consistent with the typical mode of action of antimicrobial peptides and other secreted metabolites, which exert bactericidal effects by damaging cell structures and inducing intracellular content leakage.

2. Leakage of intracellular nucleic acids and proteins

The cell membrane serves as a critical barrier that maintains structural integrity. Upon membrane disruption, intracellular proteins and nucleic acids are released into the extracellular environment. Therefore, membrane permeability is a critical indicator of cell membrane integrity. Figure 6c,d illustrate changes in nucleic acid and protein content in *L. monocytogenes* treated with postbiotics. The bacterial supernatant from the PBS buffer treatment group showed no significant changes in nucleic acid or protein levels. However, treatment with postbiotics significantly increased the release of intracellular nucleic acids and proteins in *L. monocytogenes*. At 2× MIC, the absorbance values corresponding to released nucleic acids and proteins were significantly higher than those observed in the 1/2× MIC treatment group and the untreated control. These results indicate that higher concentrations of postbiotics can significantly disrupt the structural integrity of the cell membrane, leading to the leakage of intracellular components, increased membrane permeability, and impaired cellular homeostasis. Collectively, the result suggests that the antibacterial activity of the postbiotics against *L. monocytogenes* is associated, at least in part, with membrane-associated damage, leading to leakage of intracellular nucleic acids and proteins.

3. Analysis of membrane depolarization

To further investigate whether the postbiotics affect the cytoplasmic membrane of *L. monocytogenes*, membrane depolarization was assessed using the potential-sensitive fluorescent probe DISC_3_(5). As shown in Figure 6e, fluorescence intensity increased progressively with increasing concentrations of postbiotics compared to the untreated control, indicating a dose-dependent effect. Kinetic curve analysis revealed that at 1/2× MIC induced only partial membrane depolarization, whereas exposure to 2× MIC and 4× MIC, membrane potential was nearly dissipated, suggesting disruption of energy metabolism and ionic homeostasis. These results indicate that postbiotics of *B. velezensis* 906 may inhibit bacterial growth by impairing the proton motive force and energy metabolism. It is noteworthy that DiSC_3_(5) reflects changes in membrane potential rather than direct structural integrity. Therefore, complementary assays, such as PI staining, are necessary to confirm specific structural targets of the postbiotics.

#### 3.5.3. Effects of Fermentation Supernatant on Cell Morphology of *L. monocytogenes*

1. Morphological observation by SEM and AFM

**Figure 7:**
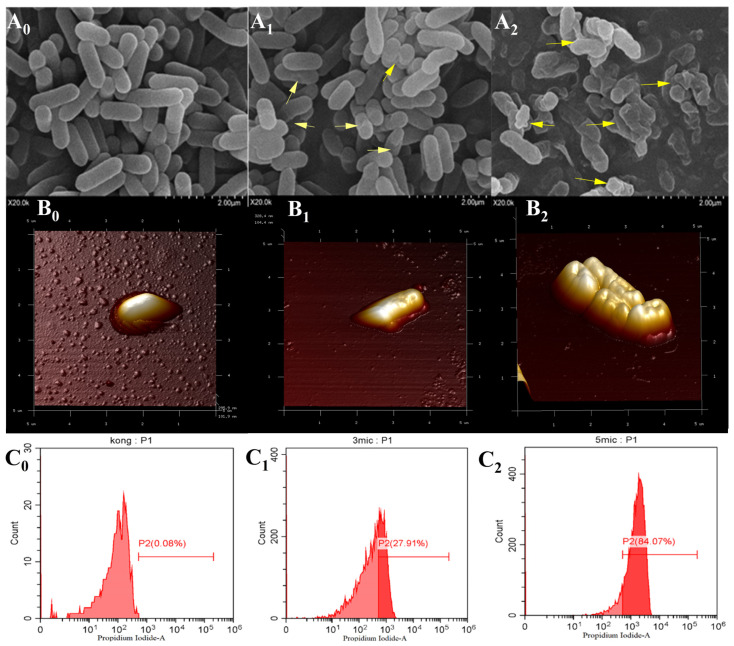
Morphological alterations and membrane damage of *L. monocytogenes* after treatment. (**A_0_**–**A_2_**), scanning electron microscopy images of untreated cells (**A_0_**), cells treated for 2 h (**A_1_**), and cells treated for 10 h (**A_2_**). (**B_0_**–**B_2_**), atomic force microscopy images of untreated cells (**B_0_**) and treated cells showing progressive surface damage (**B_1_**,**B_2_**). (**C_0_**–**C_2_**), flow cytometry analysis of membrane integrity in untreated cells (**C_0_**) and cells treated at 3× MIC (**C_1_**) and 5× MIC (**C_2_**), respectively.

The effects of the *B. velezensis* postbiotics on the morphology of *L. monocytogenes* were examined using SEM and AFM (Figure 7(A_0_,A_1_,A_2_,B_0_,B_1_,B_2_)). The results revealed that the treatment induced pronounced structural damage to the bacterial cell membrane. Cells exhibited smooth and intact surfaces with a typical rod-shaped morphology, indicating normal physiological status. Following 2 h of treatment, partial surface depressions and membrane roughening were observed, suggesting early disruption of membrane integrity. With prolonged treatment up to 10 h, extensive cellular damage became evident, including pronounced collapse, shrinkage, and eventual cell lysis. AFM imaging corroborated these observations, revealing severe distortion and collapse of the cell surface architecture, accompanied by leakage of intracellular contents, indicating aggravated membrane damage. These ultrastructural changes are consistent with flow cytometry results, which showed increased membrane permeability in a time- and dose-dependent manner. Collectively, the SEM, AFM, and PI staining results suggest that the postbiotics may impair membrane integrity and increase membrane permeability in *L. monocytogenes*.

2. Flow cytometry analysis

Flow cytometry combined with PI staining was employed to evaluate the effects of *B. velezensis* 906 postbiotics at different concentrations on the membrane integrity of *L. monocytogenes* (Figure 7(C_0_,C_1_,C_2_)). In the untreated control group, only a minimal proportion (0.08%) of cells showed PI uptake, indicating intact membranes integrity and a normal physiological function. When treated with postbiotics at 3 × MIC, the proportion of PI-positive cells increased significantly to 27.91%, demonstrating remarkable changes in membrane permeability and partial cell inactivation. Notably, treatment with 5 × MIC resulted in 84.07% of cells showing PI-positive signals, confirming extensive membrane damage and substantial bacterial inactivation. In summary, a clear concentration-dependent correlation was observed between the application of postbiotics and the disruption of cell membrane integrity, highlighting their potent bactericidal activity against *L. monocytogenes*.

#### 3.5.4. Effects of Cell Wall and Membrane Components on the Antibacterial Activity of Postbiotics

**Figure 8:**
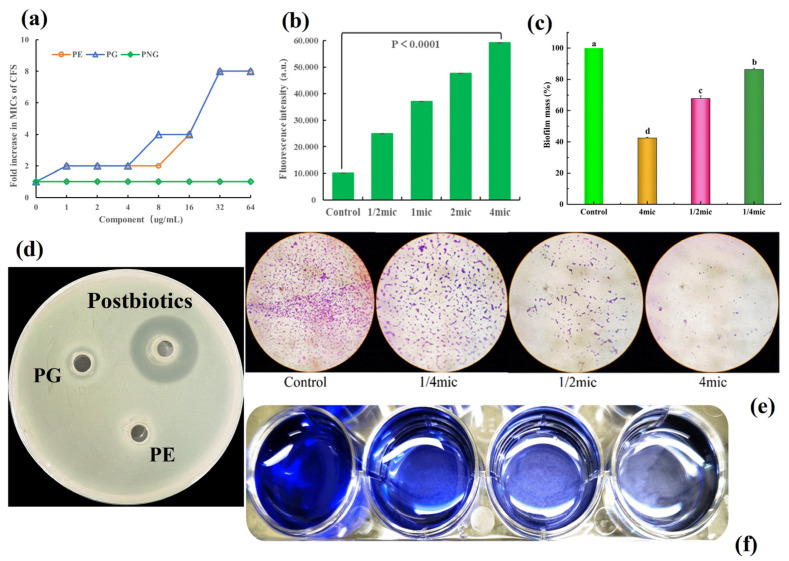
(**a**) Effects of exogenous addition of PG, PE and PGN (0–64 μg/mL) on the antibacterial activity of postbiotics. (**b**) Intracellular ROS detection by DCFH-DA staining after postbiotics treatment. (**d**) Image of inhibition zone test by coincubating postbiotics with 64 μg/mL of exogenous PE and PG against *L. monocytogenes*. (**c**) Quantitative and qualitative (**e**,**f**) analyses were employed to evaluate the effects of *B. velezensis* 906 postbiotics on *L. monocytogenes* biofilm formation. Different lowercase letters indicate significant differences (*p* < 0.05).

To elucidate whether the postbiotics exert antibacterial activity through membrane targeting, competitive binding experiments were conducted. Varying concentrations (0–64 μg/mL) of phospholipid components (PG and PE), as well as the cell wall component PGN, were added exogenously to evaluate their effects on antimicrobial activity against *L. monocytogenes*. As shown in Figure 8a, the addition of PGN did not significantly affect antibacterial activity, indicating no significant interaction between the postbiotics and the primary cell wall component PGN. In comparison, at low concentrations of PG and PE, the antibacterial activity remained largely unchanged. However, as the concentrations of these phospholipids gradually increased, the antimicrobial efficacy of the postbiotics progressively decreased. When the phospholipid concentration reached 64 μg/mL, the antimicrobial activity decreased to 1/8 of its original level. Furthermore, the inhibition zone assay also indicated that PE and PG would be the main target components of the postbiotics (Figure 8d). These findings suggest that the postbiotics may exert antibacterial effects by specifically binding to various phospholipid molecules on bacterial cell membranes, thereby disrupting membrane structure through interactions at multiple target sites. This result is consistent with the findings of Cheng et al. (2022) [30], which also demonstrated that antimicrobial components can penetrate bacterial membranes through high-affinity binding to membrane phospholipids as a universal mechanism.

#### 3.5.5. Effects of the *B. velezensis* 906 Postbiotics on Intracellular ROS Levels in *L. monocytogenes*

ROS are critical intermediates generated during aerobic metabolism. However, excessive ROS accumulation can overwhelm cellular antioxidant defenses, resulting in oxidative stress characterized by lipid peroxidation, protein modification, and DNA damage, ultimately impairing membrane integrity and cellular homeostasis. To investigate whether the postbiotics of *B. velezensis* 906 induces oxidative stress, intracellular ROS levels in *L. monocytogenes* were measured following treatment with the postbiotics at 1/2× MIC, 1× MIC, 2× MIC, and 4× MIC. The results showed that ROS levels increased significantly in a concentration-dependent manner compared with the untreated control (Figure 8b). In the control group, fluorescence intensity remained minimal, whereas even at 1/2× MIC, ROS levels were significantly elevated, indicating that low concentrations were sufficient to induce oxidative stress. Further increases in postbiotics concentration resulted in progressive ROS accumulation, reaching maximal levels at 4× MIC. These findings suggest that the bioactive components of the postbiotics not only directly disrupt the bacterial membrane integrity but also induce excessive intracellular ROS accumulation, thereby accelerating oxidative damage and facilitating bacterial cell death. Collectively, the results indicate that the postbiotics of *B. velezensis* 906 exert potent bactericidal activity through a membrane-targeting mechanism accompanied by oxidative stress induction.

#### 3.5.6. Effects of the Postbiotics of *B. velezensis* 906 on Biofilm Formation by *L. monocytogenes*

Biofilms are structured barriers formed by microbial extracellular polymeric substances, which provide physical protection through a macromolecular matrix and significantly enhance bacterial tolerance to environmental stresses and antimicrobial agents [31]. As shown in Figure 8c,e,f, *L. monocytogenes* formed dense biofilms on glass surfaces after 24 h of incubation. Treatment with 1/4× MIC of the postbiotics notably reduced bacterial adhesion, while 1/2× MIC resulted in sparsely distributed and structurally disrupted biofilms. At 4× MIC, only minimal surface-associated cells were observed, indicating substantial inhibition of biofilm establishment. Quantitative assessment of biofilm inhibition was performed by measuring absorbance at 570 nm. The control group exhibited the highest absorbance, suggesting the formation of a complete and stable biofilm. In contrast, absorbance values decreased progressively with increasing postbiotics concentration. In particular, biofilm formation was almost completely suppressed at 4× MIC, with absorbance values significantly lower than that of the control (*p* < 0.01), demonstrating a clear concentration-dependent inhibitory effect. The inhibition of biofilm formation observed in this study is likely associated with the membrane-targeting activity of the postbiotics. Biofilm development depends on early surface adhesion, intercellular communication, membrane homeostasis, and extracellular matrix production. Therefore, the membrane disruption caused by the postbiotics may impair not only bacterial viability but also the physiological processes required for stable biofilm formation. In addition, the increased intracellular ROS level induced by the postbiotics may further aggravate cellular stress, thereby interfering with biofilm maturation and structural maintenance. Taken together, these results indicate that *B. velezensis* 906-derived postbiotics effectively inhibit biofilm formation by *L. monocytogenes*, likely through combined effects on membrane integrity, oxidative stress, and biofilm-associated physiological functions, highlighting their potential application value for food preservation and pathogen control.

### 3.6. Non-Targeted Metabolomics Analysis of Postbiotics from B. velezensis 906

**Figure 9:**
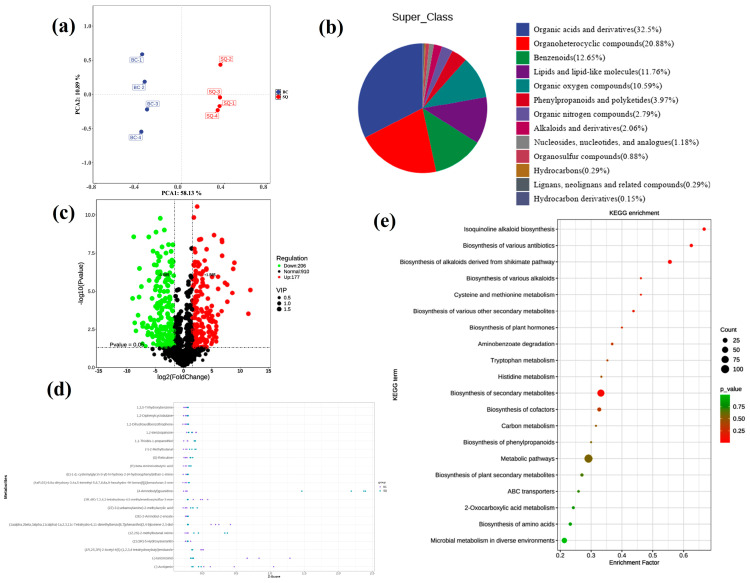
Metabolomic Analysis Between BC and SQ Groups. (**a**) PCA of group segregation. (**b**) Chemical superclass annotation of differential metabolites. (**c**) Volcano plot of metabolic changes. (**d**) Z-score based clustering pattern of differential metabolites. (**e**) KEGG enrichment analysis of differential metabolites.

Principal component analysis (PCA) was performed to evaluate metabolic differences between samples collected before and after fermentation (BC and SQ groups). As shown in Figure 9a, samples from the two groups were clearly separated into distinct quadrants, indicating significant differences in their metabolite profiles. Tight clustering within each group further demonstrated good reproducibility and significant metabolic divergence induced by fermentation. At the chemical superclass level (Figure 9b), postbiotics from *B. velezensis* 906 were predominantly concentrated in organic acids and their derivatives, accounting for the highest proportion (32.5%). This finding indicated that the strain produced large amounts of small-molecule organic acids during fermentation. These compounds not only participated in carbon flow metabolism and energy conversion but may also directly inhibit pathogen growth by lowering environmental pH. The proportions of heterocyclic organic compounds (20.88%) and phenolic compounds (12.65%) were also abundant, suggesting that the postbiotics contained abundant aromatic and nitrogen-containing heterocyclic small molecules, which are often associated with secondary metabolite activity and antimicrobial effects. In addition, the presence of lipids and lipid-like molecules (11.76%) and organically oxidized compounds (10.59%) reflected the metabolic activity of the strain in membrane biosynthesis and redox homeostasis. Although phenylpropanoids and polyketides (3.97%), organic nitrogen compounds (2.79%), alkaloid derivatives (2.06%), and nucleosides (1.18%) were present at lower proportions, their potential biological functions should not be overlooked, as they may play significant roles in antibacterial activity and signaling regulation. In summary, the metabolite profile of *B. velezensis* 906 fermentation broth exhibits a diversified composition dominated by organic acids, heterocyclic compounds, and aromatic derivatives, suggesting that its antibacterial effects likely originate from the synergistic action of multiple metabolites.

Differential metabolites were identified based on variable importance in projection (VIP > 1), fold change (FC ≥ 3 or FC ≤ 1/3), and *p* < 0.05 (Supplementary Table S3). The significant increase in differential metabolites in the fermentation medium indicated that *B. velezensis* secretes multiple secondary metabolites. Volcano plot and Z-score analyses (Figure 9c,d) identified a total of 177 upregulated and 206 downregulated differential features. Differences in metabolite abundance were observed between the control and postbiotics groups. Overall, the postbiotics group exhibited higher Z-score values for most metabolites, suggesting their enrichment or enhanced activity in the fermentation supernatant. In particular, aromatic compounds such as 1,3,5-trihydroxybenzene, 1,2-benzoquinone, and 1,2-dihydroxydibenzothiophene were markedly enriched in the postbiotics, suggesting enhanced redox activity that may contribute to oxidative stress induction and membrane disruption in *L. monocytogenes*. Additionally, sulfur- and nitrogen-containing metabolites, including 1,1-thiobis-1-propanethiol, 4-aminobutylguanidine, and (2E)-2-aminobut-2-enoate, were also more abundant, implying that amino acid decarboxylation and sulfur metabolism were markedly activated during fermentation. These metabolites are often associated with antimicrobial or quorum-sensing regulatory effects. Moreover, bioactive alkaloids and phenolic derivatives such as (S)-reticuline, (–)-lariciresinol, and (–)-arctigenin were detected at much higher levels in the postbiotics, consistent with their known antimicrobial and antioxidant properties; this finding provides a potential mechanistic explanation for how these metabolites inhibited *L. monocytogenes* viability through oxidative stress regulation. These findings suggest that fermentation by *B. velezensis* 906 substantially remodels the extracellular chemical environment and generates a set of candidate bioactive metabolites that may contribute to the observed anti-*Listeria* effect.

To further investigate the pathways associated with differential metabolites, KEGG annotation was performed on postbiotics secreted by *B. velezensis* 906 to identify the most prominent and crucial metabolic and biosynthetic pathways (Figure 9e). The results revealed significant enrichment in the bioactive isoquinoline alkaloid biosynthesis pathway, the shikimic-derived alkaloid synthesis pathway, and biosynthesis pathways of multiple antibacterial compounds. These findings suggest that *B. velezensis* 906 may activate multiple metabolic pathways associated with structurally diverse bioactive molecules during fermentation. In particular, the enrichment of amino acid metabolic pathways may be relevant not only to central metabolic adaptation but also to the biosynthesis of peptide- or alkaloid-related antibacterial compounds. Collectively, the untargeted metabolomics supports the notion that the antibacterial activity of *B. velezensis* 906-derived postbiotics is underpinned by a chemically complex metabolite system, thereby providing a metabolite-level basis for the strong inhibitory phenotype observed against *L. monocytogenes*.

## 4. Discussion

*L. monocytogenes*, a common foodborne pathogen and spoilage microorganism, contaminates a wide range of foods and poses a serious threat to food safety and public health. Previous studies have shown that natural antimicrobial compounds play a crucial role in controlling foodborne pathogens, preventing contamination, improving food safety, and enhancing the sensory quality of food [32]. Bacterial-derived metabolites, particularly postbiotics, have proven to be highly effective natural preservatives. *Bacillus* species have received significant attention for their ability to synthesize a wide range of functional postbiotics. These compounds confer strong antibacterial properties, enabling *Bacillus* strains to effectively inhibit various microorganisms, including Gram-positive bacteria, Gram-negative bacteria, and fungi [33]. Previous reports have shown that strains such as *B. subtilis* and *B. licheniformis* exhibit pronounced inhibitory activity against *L. monocytogenes* [34]. In this study, a *Bacillus* strain capable of secreting antibacterial metabolites was isolated and screened from the rice substrate. Based on 16S rRNA gene sequencing and whole-genome mining analysis, it was identified as a Gram-positive bacterium and designated *B. velezensis* 906. In recent years, the advancement of whole-genome sequencing technologies and bioinformatic mining tools has revealed the widespread presence of numerous biosynthetic gene clusters (BGCs) related to antimicrobial activity in various *Bacillus* species. Whole-genome mining revealed that *B. velezensis* 906 harbors multiple antimicrobial-associated BGCs, including surfactin, fengycin, bacillaene, macrolactin, difficidin, bacillibactin, subtilin, and bacilysin. This finding is consistent with recent reports on antimicrobial secondary metabolites produced by *Bacillus* spp. [35,36] and highlights the potential of strain 906 as an efficient antagonistic bacterium. It also provides a theoretical basis for understanding the diversity and possible synergistic interactions underlying its antimicrobial activity. As representative secondary metabolites of *Bacillus*, lipopeptides including surfactin and fengycin are encoded by highly conserved BGCs in strain 906. Mnif et al. reported that both surfactin and fengycin are cyclic lipopeptides synthesized by NRPSs and that their amphiphilic architecture is fundamental to their bioactivity [37]. Surfactin exerts antibacterial effects by disrupting target microbial membrane integrity and causing intracellular leakage; its antimicrobial efficacy may often be enhanced when acting in concert with other bioactive compounds, which is consistent with the findings of Sudarmono et al. (2019), who reported that surfactin alone exhibited relatively weak antimicrobial activity but showed improved efficacy when combined with fengycin [38]. In contrast, fengycin shows potent antifungal activity against plant pathogenic and filamentous fungi, likely by interacting with sterol-containing fungal membranes to form transmembrane pores and accelerate cell death [39]. Polyketides represent another important class of antimicrobial metabolites in *Bacillus* spp. In the present study, the bacillaene-, macrolactin-, and difficidin-related BGCs identified in strain 906 belonged to PKS or hybrid NRPS–PKS gene clusters, and the compounds encoded by these clusters are known to exhibit broad antimicrobial spectra with distinct modes of action. For example, bacillaene has been reported to inhibit bacterial fatty acid biosynthesis, thereby interfering with membrane formation and repair, as described by Arguelles-Arias et al. (2025) in *B. amyloliquefaciens* GA1 [40]. Additionally, strain 906 also carried conserved BGCs related to bacillibactin, subtilin, and bacilysin, which likely serve complementary roles in the antimicrobial metabolic network of *Bacillus* [41]. Among them, Subtilin is a ribosomally synthesized and post-translationally modified peptide that mainly acts by disrupting bacterial membrane integrity and forming transmembrane pores, thereby causing osmotic imbalance and cell death [42]. Overall, the antimicrobial-associated BGCs harbored by *B. velezensis* 906 cover diverse categories, including lipopeptides, polyketides, siderophores, RiPPs, and simple peptides. The antimicrobial metabolites encoded by these BGCs may exert distinct antimicrobial mechanisms, which could effectively broaden the antimicrobial spectrum, improve antimicrobial efficacy, and lower the risk of resistance evolution in target microorganisms. Collectively, these features suggest a potential advantage of *Bacillus* strains over microorganisms that rely primarily on a single antimicrobial compound. The coexistence of these well-characterized clusters indicates that strain 906 retains the canonical capacity of *Bacillus* species to synthesize structurally diverse antimicrobial metabolites. Compared with previously reported strains possessing only limited subsets of lipopeptide-associated BGCs (e.g., surfactin–fengycin or macrolactin–bacillaene combinations) [43,44], strain 906 exhibits a broader biosynthetic repertoire encompassing NRPS-, PKS-, RiPP-, and metallophore-related systems. Such genomic architecture suggests substantial biosynthetic potential and possible functional complementarity among antimicrobial-related pathways. Notably, in addition to these high-confidence conserved clusters, several type II/type III PKS- and terpene-associated BGCs lacking close matches in the MIBiG database were also identified. Although the products of these orphan clusters remain uncharacterized, related PKS and terpene systems have been reported to generate aromatic polyketides, quorum-sensing modulating, or hydrophobic compounds capable of influencing membrane properties. These features imply that strain 906 may possess additional, yet uncharacterized biosynthetic potential. However, the specific products and biological roles of these orphan clusters remain unclear and require further validation. Collectively, rather than representing a single lipopeptide-driven antibacterial model, strain 906 is likely to establish a multilayered antimicrobial network integrating membrane disruption (lipopeptides), intracellular biosynthetic interference (polyketides), iron competition (metallophores), and potential regulatory modulation. Such a multi-component and multi-pathway system provides a plausible explanation for the pronounced inhibitory activity of its postbiotics against *L. monocytogenes* and may reduce the likelihood of rapid resistance development associated with single-target antimicrobial agents [45]. In addition, inhibition assays revealed that the postbiotics produced by strain 906 exhibited strong inhibitory activity against Gram-positive bacteria, consistent suppression effects on Gram-negative bacteria, and variable inhibition toward fruit spoilage microorganisms. The antimicrobial spectrum observed in this study appeared broader than that reported for many previously described bacterial metabolite preparations, which generally exhibit strong inhibition of Gram-positive bacteria but weak activity against Gram-negative bacteria [46]. Furthermore, in combination with the short ribosomally derived antimicrobial peptides identified in this study, strain 906 displays a dual metabolic configuration characterized by the coexistence of short cationic peptides and NRPS/PKS-derived secondary metabolites [47]. This integrated metabolic profile may represent a useful metabolite-rich postbiotic resource with potential relevance for food safety applications.

*Bacillus* strains generally possess the genetic capacity to form multicomponent antimicrobial metabolic networks. Previous studies have shown that whole-genome sequence analysis of *B. velezensis* TJS119 allowed the identification of several biosynthetic gene clusters putatively associated with antifungal activity, and subsequent UPLC-MS/MS analysis led to the detection of fengycin [48]. Furthermore, Chen et al. (2025) reported that *B. velezensis* B115 harbored 13 BGCs in its genome, together with a complex metabolite profile in its fermentation broth [49]. Collectively, these studies imply that there may be a certain degree of consistency between BGC composition at the genomic level and the extracellular chemical profiles detected by metabolomics among members of the genus *Bacillus*. In the present study, genome mining revealed that strain 906 harbors multiple biosynthetic gene clusters related to lipopeptides, polyketides, RiPPs, siderophores, and other bioactivity-associated metabolites, indicating broad biosynthetic potential. Meanwhile, untargeted metabolomics demonstrated substantial remodeling of the extracellular metabolite composition after fermentation and suggested the accumulation of multiple candidate antimicrobial-related metabolites. When considered together, these results support the hypothesis that the anti-*Listeria* activity of *B. velezensis* 906-derived postbiotics is mediated by a multi-component antimicrobial system rather than by a single metabolite alone. Such complexity in metabolite composition and function reasonably explains the remarkable antibacterial activity of these postbiotics and establishes a system-level rationale for the subsequent observations of membrane damage, oxidative stress, and biofilm inhibition. However, these association analyses based on genomics and metabolomics still present certain limitations. First, the BGCs predicted by genome mining only indicate the putative biosynthetic capacity of the strain, and do not confirm that the corresponding metabolites are expressed and secreted under the tested culture conditions. Previous studies have indicated that many microbial BGCs may be silent or poorly expressed under conventional laboratory conditions. Thus, genomic data alone cannot sufficiently verify that the relevant metabolites are actually produced and participate in the detected antimicrobial activity [50]. Second, although untargeted LC–MS metabolomics can reveal the differences in extracellular metabolites, metabolite annotation remains limited by identification bottlenecks. Without authentic standards and high-quality MS/MS spectra for rigorous validation, most annotated metabolites can be assigned only tentative identities [51]. Accordingly, strain 906 is inferred to harbor the potential to produce various candidate antimicrobial metabolites, and its antibacterial activity likely arises from the synergistic effects of multiple components. However, the precise sources, biological functions, and actual contributions of these metabolites to the antimicrobial mechanism still require further targeted validation.

**Figure 10:**
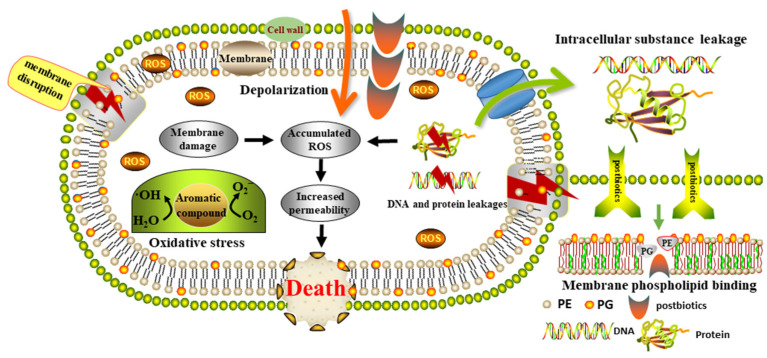
Antibacterial mechanism of metabolites against *L. monocytogenes*.

The bacterial cell membrane not only maintains cellular homeostasis but also plays essential roles in vital biological processes such as ion transport, compound exchange, and osmoregulation. As the primary barrier against environmental stress, membrane integrity is indispensable for bacterial survival [52]. Previous studies have shown that postbiotics can directly target bacterial membranes by disturbing phospholipid bilayer organization, thereby inhibiting the growth and proliferation of pathogenic microorganisms. In this study, metabolites derived from the postbiotics of *B. velezensis* 906 exhibited significant antibacterial activity against *L. monocytogenes*. The experimental findings suggested that the postbiotics derived from *B. velezensis* 906 exerted antibacterial activity against *L. monocytogenes* predominantly through a membrane-targeting mechanism. The bacterial cell membrane is essential for maintaining cellular homeostasis and plays critical roles in ion transport, compound exchange, and osmoregulation. As the primary barrier against environmental stress, its structural integrity is indispensable for bacterial survival. In the present study, the increased AKP activity and enhanced leakage of intracellular nucleic acids and proteins indicated substantial disruption of the cell envelope barrier. This interpretation was further supported by the rapid dissipation of membrane potential detected by DiSC_3_(5), the significant increase in PI-positive cells, and the severe surface collapse and distortion observed by SEM and AFM. Furthermore, previous studies have demonstrated that postbiotics can directly target bacterial membranes by disturbing the organization of phospholipid bilayers, which is consistent with the membrane-targeting mechanism observed in this study. Importantly, phospholipid competition assays suggest that membrane phospholipids, particularly PG and PE, are relevant interaction targets whereas PGN does not appear to be a major target, indicating that the postbiotics primarily impair cytoplasmic membrane homeostasis, leading to loss of permeability control, membrane depolarization, intracellular leakage, and ultimately bacterial inactivation, and in parallel, the concentration-dependent accumulation of intracellular ROS suggests that oxidative stress acts as an additional aggravating factor, which further accelerates membrane-associated damage and cellular dysfunction, consistent with Li et al. (2023), who reported that postbiotics from Lactobacillus plantarum FB-2 induced the accumulation of endogenous ROS in *S. aureus*, leading to leakage of cellular contents [53]. Collectively, these findings suggest that the antibacterial mechanism of postbiotics produced by *B. velezensis* 906 may be associated with membrane phospholipids, which may contribute to the disruption of membrane structure, the disturbance of key physiological processes in *L. monocytogenes*, and ultimately lead to bacterial cell death (Figure 10).

The postbiotics produced by *B. velezensis* 906 exhibited significant antimicrobial activity, and their related BGCs involve multiple metabolic pathways, including lipopeptides and polyketides. Therefore, these postbiotics show promising application potential in food systems. Previous studies have demonstrated that microbial-derived postbiotics can serve as natural food preservatives to inhibit foodborne pathogens and spoilage microorganisms such as *L. monocytogenes*, thereby extending the shelf life of perishable foods including dairy, meat, fruit, and vegetable products [54]. Nevertheless, limitations of the present study should be acknowledged. Although the integrated genomic and metabolomic analyses provided important system-level evidence for the antibacterial basis of the postbiotics, the reported metabolites remain putatively annotated rather than unequivocally identified, and the contribution of each individual compound was not experimentally validated after purification. Beyond this, while the combined phenotypic and competition assays strongly support a membrane-targeted antibacterial mechanism, the precise molecular binding modes between specific postbiotic components and membrane phospholipids remain to be clarified. Therefore, future studies should focus on targeted isolation and structural confirmation of key active compounds, as well as mechanism-specific validation to define the principal antibacterial determinants of *B. velezensis* 906-derived postbiotics. Furthermore, the active components potentially present in the postbiotics, such as surfactin and fengycin, may contribute to improving the microbial safety and quality stability of foods. However, further studies are needed to optimize the production, purification, and stability of these postbiotics under different food processing conditions and to evaluate their safety and efficacy in actual food matrices, which will lay a solid foundation for their practical application in the food industry.

## 5. Conclusions

In conclusion, postbiotics from *B. velezensis* 906 showed strong antibacterial activity, especially against *L. monocytogenes*, achieving an MIC of 0.0083 mg/mL, indicating their potential as natural antimicrobial agents. Their antibacterial effect was associated with membrane-related damage and oxidative stress. Genome mining combined with non-targeted metabolomics suggested that this activity may arise from a coordinated multi-component antimicrobial system. The differential metabolites were mainly enriched in terpenoid- and polyketide-related and aromatic structural classes. Overall, these findings provide a basis for further development of *B. velezensis* 906 postbiotics for food safety applications.

## Data Availability

The original contributions presented in this study are included in the article/Supplementary Materials. Further inquiries can be directed to the corresponding authors.

## Supplementary Materials

The following supporting information can be downloaded at https://www.mdpi.com/article/10.3390/foods15081364/s1. Table S1: Strain information; Table S2: Genome features of B. velezensis 906; Table S3: The list and information of differential accumulation metabolites identified in this study.

## Abbreviations

The following abbreviations are used in this manuscript:

NRPSs	nonribosomal peptide synthetases
PKSs	polyketide synthases
BGCs	biosynthetic gene clusters
ATCC	American Type Culture Collection
LB	Luria–Bertani
TSB	tryptic soy broth
PDB	potato dextrose broth
KEGG	Kyoto Encyclopedia of Genes and Genomes
RefSeq	reference sequence database
COGs	clusters of orthologous groups
PBS	phosphate-buffered saline
MIC	minimum inhibitory concentration
CLSI	Clinical and Laboratory Standards Institute
AKP	alkaline phosphatase
PI	propidium iodide
SEM	scanning electron microscopy
AFM	atomic force microscopy
ROS	reactive oxygen species
PGN	peptidoglycan
PG	phosphatidylglycerol
PE	phosphatidylethanolamine
LC-MS	liquid chromatography–mass spectrometry
HMDB	Human Metabolome Database
ANOVA	analysis of variance
MIBiG	minimum information about a biosynthetic gene cluster
PCA	principal component analysis
