# Supplementary material for: Integrated Metabolomic and Genomic Analysis of the Antibacterial Mechanism of Postbiotics Derived from Bacillus velezensis 906 Against Listeria monocytogenes
Source: Foods. 2026 Apr 14;15(8):1364. doi: 10.3390/foods15081364 (PMC13115427; doi:10.3390/foods15081364)
Supplement: Supplementary file 1 [file foods-15-01364-s001.zip › foods-4205208-supplementary.pdf]

Integrated metabolomic and genomic analysis of the antibacterial mechanism of postbiotics derived from *Bacillus velezensis* 906 against *Listeria monocytogenes*

Zhe Liu [a], Xuetuan Wei [a], Qingyan Pan [a], Xifeng Zuo [a], Ping Chen [b, *], Ailing Guo [a,*]

a College of Food Science and Technology, Huazhong Agriculture University, Wuhan 430070, China.

b Suizhou Center for Disease Control & Prevention, Suizhou, Hubei, 441300, China

* Corresponding author.

E-mail address: guoailing@mail.hzau.edu.cn (A. Guo).

chenp3976@163.com (P. Chen).

Supplementary Tables

Table S1 Strain information

Strain	Medium	Culture conditions
Bacillus velezensis 906	LB	37 ℃, aerobic, 28 h
Listeria monocytogenes	TSB	37 ℃, aerobic, 24 h
Escherichia coli	LB	37 ℃, aerobic, 24 h
Staphylococcus aureus	LB	37 ℃, aerobic, 24 h
Cronobacter sakazakii	TSB	37 ℃, aerobic, 24 h
Colletotrichum acutatum	PDB	25 ℃, aerobic, 96 h
Colletotrichum gloeosporioides	PDB	25 ℃, aerobic, 96 h
Fusarium oxysporum	PDB	25 ℃, aerobic, 96 h
Salmonella enteritidis	LB	37 ℃, aerobic, 24 h
Salmonella muenchen	LB	37 ℃, aerobic, 24 h
Bacillus cereus	LB	37 ℃, aerobic, 24 h

Table S2 Genome features of *B. velezensis* 906.

Genomic Feature	Value
Size of the genome assembly (bp)	3,954,104
GC content (%)	46.64
Protein-coding genes / regions (bp)	3,809 / 3,504,309
tRNA / 16S rRNA genes	86 / 9
Genes assigned to COG categories	2988
CRISPR	0
Genomic island	0

Table S3 The list and information of differential accumulation metabolites identified in this study.

Index	Metabolite	VIP	Pvalue	Fold_Change	type
M001T0002	(-)-N-(2S,3R)-3-(Amino-2-hydroxy-4-(4-hydroxyphenyl)butyryl)-L-leucine	1.471649	0.000149	4.003205834	up
M001T0032	N-Acetyl-D-phenylalanine	1.448266	0.000876	80.60206275	up
M001T0072	19-Hydroxy-royleanone	1.421729	0.002583	1560564.729	up
M001T0086	7,8 Dihydrokawain	1.482072	7.23E-06	11.03576915	up
M001T0091	(2E)-3-[4-hydroxy-3-(sulfooxy)phenyl]prop-2-enoic acid	1.430472	0.002265	4.027907897	up
M001T0135	methyl (4R,4aS)-4-(acetyloxy)-8-hydroxy-6-methyl-9-oxo-4a,9-dihydro-4H-xanthene-4a-carboxylate	1.41469	0.003422	7.31928146	up
M001T0145	Suberic acid	1.410663	0.004038	6.951800022	up
M001T0178	Penihydrone	1.408061	0.003543	3.188516259	up
M001T0182	Khelmarin D	1.432341	0.001938	1492441	up
M001T0189	Geranyl beta-D-glucopyranoside	1.404685	0.004362	8.101649051	up
M001T0218	N1,N5,N10-Tris-trans-p-coumaroylspermine	1.438298	0.001273	3.322786929	up
M001T0239	5-nitro-2-propoxyaniline	1.398256	0.005403	19.24212697	up
M001T0265	Mayzent	1.4834	6.40E-06	36.93393275	up
M001T0269	Azadirachtin P	1.420202	0.003189	6.194883745	up
M001T0283	Estrone 3-glucuronide	1.47934	2.24E-05	1383542	up
M001T0291	5-Cholesten-3beta-25-diol-3-sulfate	1.447299	0.001094	7.815713639	up
M001T0327	2H-Pyran-2-one, 4-(fluoromethyl)tetrahydro-4-hydroxy-	1.40537	0.004259	17.75473357	up
M001T0343	Cladospolide B	1.478582	4.27E-05	8.121777032	up
M001T0372	Chitotriose	1.447593	0.000926	11.40864331	up
M001T0382	Dihydronaringenin-O-sulphate	1.448262	0.000985	3.757276029	up
M001T0387	2-hydroxy-8-[(2S)-1-(2-hydroxybenzoyl)pyrrolidine-2-carbonyloxy]tetradecanimidic acid	1.45994	0.000341	4.213187832	up

M001T0418	2-(2-carboxyethyl)-4-methyl-5-propylfuran-3-carboxylic acid	1.482377	1.30E-05	15.96389113	up
M001T0435	7-Hydroxyemodin	1.4525	0.000759	506669.4	up
M001T0440	(E)-7-hydroxyhept-2-enoic acid	1.413701	0.00329	46.89927138	up
M001T0442	Aspernigrin B	1.458978	0.000535	11.20559393	up
M001T0466	PE(12:0/0:0)	1.416785	0.002871	3.360585554	up
M001T0481	3-(3,4-dimethoxyphenyl)-5-hydroxy-7-methoxy-8-methyl-4H-chromen-4-one	1.439294	0.001348	5.595331345	up
M001T0583	N-(1-Deoxy-1-fructosyl)valine	1.450167	0.000753	21.8713289	up
M001T0584	N-(1-Deoxy-1-fructosyl)tyrosine	1.456907	0.000644	122389.6	up
M001T0593	L-gamma-Glutamyl-S-allylthio-L-cysteine	1.433874	0.002225	3.37057373	up
M001T0627	Kaempferol 3-[4-(p-coumaroylglucosyl)rhamnoside]	1.439257	0.001361	9.075281737	up
M001T0630	Physapruin B	1.423464	0.002699	9.216959039	up
M001T0639	8-Deoxy-11-hydroxy-13-chlorogrosheimin	1.400338	0.004753	7.123952483	up
M001T0650	Aeglin	1.419585	0.002876	53.44491933	up
M001T0676	[5,7-dihydroxy-2-(4-hydroxyphenyl)-4-oxo-3,4-dihydro-2H-1-benzopyran-3-yl]oxidanesulfonic acid	1.478869	3.57E-05	42.45298653	up
M001T0677	[5,6,7-trihydroxy-2-(4-hydroxyphenyl)-4-oxo-3,4-dihydro-2H-1-benzopyran-3-yl]oxidanesulfonic acid	1.429177	0.00239	287940.4	up
M001T0680	6-[5,7-dihydroxy-2-(3-hydroxy-2,2-dimethyl-3,4-dihydrochromen-6-yl)-4-oxochromen-3-yl]oxy-3,4,5-trihydroxyoxane-2-carboxylic acid	1.430592	0.001987	4.754169756	up
M001T0696	5-[(E)-2-(4-hydroxy-3-methoxyphenyl)ethenyl]-2-[(1E)-4-hydroxy-3-methylbut-1-en-1-yl]benzene-1,3-diol	1.477748	2.93E-05	16.9431771	up
M001T0706	4-[5-(3-hydroxypropyl)-1-benzofuran-2-yl]benzene-1,2-diol	1.430602	0.002425	4.097603504	up
M001T0721	3,4,5-trihydroxy-6-[(6-methyl-2-oxo-2H-chromen-3-yl)oxy]oxane-2-carboxylic acid	1.457141	0.000639	3.135804584	up
M001T0723	3,4,5-trihydroxy-6-[3-(4-methoxyphenyl)propoxy]oxane-2-carboxylic	1.453281	0.000622	61.18033299	up

	acid				
M001T0729	4-(dimethylamino)-1-(2-hydroxyphenyl)-3-methyl-2-phenylbutan-2-yl propanoate	1.485031	4.54E-07	12.20749559	up
M001T0738	Alternarizine A	1.482778	4.17E-06	16.01389656	up
M001T0754	1-(2-carboxyacetamido)cyclopropane-1-carboxylic acid	1.441227	0.001634	4.255079178	up
M001T0777	5-Formimidoyltetrahydrofolic acid	1.424648	0.002511	22.09784894	up
M001T0782	Deferitrin	1.479255	2.73E-05	249776.3	up
M001T0867	(2S)-2-[6-(acetyloxy)hexyl]-3-methylidenebutanedioic acid	1.398959	0.004945	35.48376931	up
M001T0898	Bucetin	1.421674	0.002998	9.882854513	up
M001T0925	Asacoumarin A	1.468768	0.000221	76822.18	up
M001T0944	4-Methoxy-5-(3,7,11,15-tetramethyl-2,6,10,14-hexadecatetraenyl)-1,3-benzenediol	1.416857	0.003001	5.149454219	up
M001T1006	4-Hydroxybisabola-2,10-dien-9-one	1.454713	0.000628	3.453827107	up
M001T1017	Chrysopiperazine B	1.411033	0.004099	85496.35	up
M001T1032	Scensidin	1.454041	0.000646	6.790254091	up
M001T1049	3-METHYL-2-OXINDOLE	1.441114	0.001295	8.905042592	up
M001T1080	3-(2-Furanyl)-2-propenal	1.44067	0.001454	14.82438706	up
M001T1119	Undeca-3,6,9-trienedioylcarnitine	1.478517	3.21E-05	7.911461203	up
M001T1174	PGP(PGD2/i-17:0)	1.396858	0.004931	53.66037141	up
M001T1184	PE(LTE4/22:4(7Z,10Z,13Z,16Z))	1.427544	0.002177	3.077713107	up
M001T1244	2-(5-oxidanyl-1H-indol-2-yl)ethanoic acid	1.458863	0.000463	2235387	up
M001T1301	Platyphylline N-oxide	1.457665	0.00064	13.43690085	up
M001T1312	D-Sedoheptulose 7-phosphate	1.458545	0.000574	59.17152914	up
M001T1327	Madecassoside	1.422752	0.002458	37.05612504	up
M001T1387	4-Amino-1-[(2R,3S,4S,5R)-3,4-dihydroxy-5-(hydroxymethyl)oxolan-2-yl]oxypyrimidin-2-one	1.427455	0.002414	8884976	up

M001T1394	1,7-dioxacycloheptadecan-8-one	1.396914	0.004794	12.82567732	up
M001T1419	Phenylalanylserine	1.429103	0.002379	285701.3	up
M001T1445	N-Acetyl-DL-tryptophan	1.439117	0.001366	10.03195009	up
M001T1446	1-Oxa-2-oxo-3-methylcycloheptane	1.448753	0.000847	89079.51	up
M001T1448	Tanacetin	1.471038	0.000166	7.611903658	up
M001T1462	Hyocholate	1.43799	0.00133	7.238031461	up
M001T1489	3-Acetamidobutanal	1.396151	0.005005	7.75392306	up
M001T1495	Tagitinin A	1.46593	0.00031	811851.6	up
M001T1500	Icofungipen	1.470099	0.000169	12.44013322	up
M001T1504	Ilexgenin A	1.411704	0.003143	4.11946908	up
M001T1520	mercaptohexanol	1.434921	0.001908	14.62409279	up
M001T1545	Arg-Thr-Lys-Arg	1.478935	2.52E-05	47308.94	up
M001T1550	1-indanol	1.424333	0.002201	4.640553972	up
M001T1572	Tensyuic acid B	1.397539	0.005154	7.984224868	up
M001T1573	Tensyuic acid E	1.402874	0.004309	32.35571541	up
M001T1612	Iriomoteolide 3a	1.400673	0.005118	12.3669109	up
M001T1615	Botryolide C	1.459244	0.000516	3.275484227	up
M001T1616	(5-hydroxy-2-oxo-2H-pyran-4-yl)methyl acetate	1.422442	0.002584	91.03045024	up
M001T1623	Avadomide	1.40978	0.003763	39995.67	up
M001T1633	HDMBOA-Glc	1.43959	0.00164	6.390664401	up
M001T1651	Flugestone	1.445811	0.001251	4.47718508	up
M001T1698	2-(4-hydroxyphenyl)-5,6,7,8-tetramethoxy-4H-chromen-4-one	1.47549	7.65E-05	11.08964386	up
M001T1701	3-propylbenzene-1,2-diol	1.428389	0.002035	8.545921711	up
M001T1703	3-Hydroxysebacic acid	1.46134	0.000403	7.608476728	up
M001T1707	1-(2-methylphenyl)-4-(2-{5H,6H,7H,8H-[1,2,4]triazolo[4,3-a]pyridin-3-yl}ethyl)piperazine	1.448852	0.000875	9.086676994	up

M001T1718	Dihydrotetrodecamycin	1.446198	0.000918	6.836049296	up
M001T1722	(2-oxo-2,3-dihydro-1H-indol-3-yl)acetic acid	1.424678	0.00238	5.794867495	up
M001T1730	(1)Benzopyrano(3,4-b)(1)benzopyran-12(6H)-one, 6a,12a-dihydro-2,3,9-trimethoxy-	1.447157	0.000969	11.57398277	up
M001T1752	4-(propan-2-yl)benzaldehyde	1.479922	2.08E-05	158554.8	up
M001T1762	2-(3,4,5-trimethoxyphenyl)-4H-chromen-4-one	1.485492	3.08E-07	1009226	up
M001T1768	3,4-Dimethylstyrene	1.419382	0.002763	10.14268965	up
M001T1778	11-dehydro-2,3-dinor Thromboxane B2	1.449514	0.00077	5.359627901	up
M001T1827	4-chloro-N-[2-(morpholin-4-yl)ethyl]benzamide	1.400529	0.004597	256031.4	up
M001T1857	Nitrothal-isopropyl	1.402043	0.004375	67.71248518	up
M001T1874	S-Adenosylmethioninamine	1.403281	0.004481	53.55065207	up
M001T1891	Hyaluronan biosynthesis, precursor 1	1.477069	5.26E-05	2110309	up
M001T1909	Mycothiol	1.46052	0.00038	12.54271968	up
M001T1926	Zapoterin	1.465504	0.000239	255057.3	up
M001T1969	Baptifoline	1.467511	0.000259	12.59941081	up
M001T1975	(3R,4R)-4-amino-3-[(1-carboxyeth-1-en-1-yl)oxy]cyclohexa-1,5-diene-1-carboxylic acid	1.470248	0.000164	31.49768551	up
M001T1985	2-Amino-9,10-epoxy-8-oxodecanoic acid	1.445973	0.001195	138.0980899	up
M001T1996	Xerophilusin V	1.439911	0.001678	3.579902534	up
M001T2003	Pergillin	1.447729	0.001054	11.10896957	up
M001T2006	Pseudomajucin	1.472823	0.000164	6.76945941	up
M001T2027	Phe-pro-arg	1.444675	0.000997	24.60777425	up
M001T2060	(2E)-N-(4-carbamimidamidobutyl)-3-(4-hydroxy-3-methoxyphenyl)prop-2-enamide	1.479777	4.31E-05	25.05461215	up
M001T2147	Cytosporic acid	1.421132	0.002581	3.278060362	up
M001T2153	5,7-dihydroxy-3-(4-methoxyphenyl)-4H-chromen-4-one	1.406568	0.004388	4.261098691	up

M001T2171	3,5,7-trihydroxy-2-(4-hydroxyphenyl)-4H-chromen-4-one	1.440398	0.001624	3.632776045	up
M001T2177	4,5,7-Trihydroxyisoflavone	1.445919	0.001251	3.167144785	up
M001T2203	Acteoside	1.462153	0.000359	10.11829435	up
M001T2230	11Beta-PGE2	1.404621	0.003973	3.08472967	up
M001T2233	13,14-dihydro-15-keto-tetranor PGF1Beta	1.406927	0.003712	7.98489728	up
M001T2243	1-Linoleoyl Glycerol	1.444245	0.001429	3.109321006	up
M001T2277	(2-aminoethoxy)[(2R)-2-hydroxy-3-(pentadecanoyloxy)propoxy]phosphinic acid	1.399389	0.004507	6.788632699	up
M001T2285	1-(6Z,9Z,12Z-octadecatrienoyl)-glycero-3-phosphate	1.452009	0.000849	10.22533651	up
M001T2292	Pholcodine	1.459779	0.000433	27533.45591	up
M001T2342	Decarbamoylgonyautoxin III	1.434807	0.001715	5.756336283	up
M001T2352	11-beta-Hydroxyandrosterone-3-glucuronide	1.419498	0.00264	27.21482191	up
M001T2386	(2S)-3-(2-sulfanylidene-2,3-dihydro-1H-imidazol-4-yl)-2-(trimethylazaniumyl)propanoate	1.428956	0.002261	16.38844762	up
M001T2402	3,7-Dihydroxy-3,4-dimethoxyflavone	1.468443	0.000162	4.601397472	up
M001T2406	2-amino-6-(2-hydroxypropanoyl)-2,3-dihydro-1H-pteridin-4-one	1.451908	0.000717	882.5876163	up
M001T2414	bis(2,5-dihydroxypyrrol-1-yl) octanedioate	1.409869	0.00403	196813	up
M001T2436	L-Cladinose	1.468593	0.000248	6.205433313	up
M001T2447	Chaxalactin C	1.472287	0.000143	13.88830465	up
M001T2455	4-Hydroxycoumarin	1.422133	0.002985	4.972289293	up
M001T2479	2,4-Cyclohexadien-1-ylmethanol	1.419584	0.002821	5.091299385	up
M001T2500	(2,6-dioxo-3H-purin-9-yl) pyridine-3-carboxylate	1.455713	0.00061	9.223010927	up
M001T2510	[3H]Dopamine	1.470109	0.000195	42.81897047	up
M001T2570	Robinetin trimethyl ether	1.446877	0.00111	10.52035838	up
M001T2575	1-Octyl-beta-D-glucopyranoside	1.417481	0.003421	946503.9	up
M001T2583	1,6-Anhydro-N-acetyl-beta-muramate	1.46751	0.0002	237010.4	up

M001T2602	cid_643007	1.430594	0.001951	45.81074725	up
M001T2615	(2E,6Z)-nona-2,6-dienal	1.46512	0.000275	84061.62	up
M001T2624	Salviaflaside	1.42739	0.002587	3.854574828	up
M001T2628	(6E,8E,11E,14E)-5,20-dihydroxyicosa-6,8,11,14-tetraenoic acid	1.401162	0.004692	7.666740497	up
M001T2645	Cadabicine	1.44347	0.001131	4.167636019	up
M001T2660	Isoochracinic acid	1.469694	0.000173	15.81752204	up
M001T2667	(-)-trans-C75	1.40656	0.004082	6.410668403	up
M001T2676	D41EF04C-08B7-47B6-BFC3-F0BB65679872	1.480585	1.43E-05	5.96131523	up
M001T2681	Hexamidine	1.483228	6.31E-06	277798	up
M001T2696	Dimethisterone	1.427367	0.002233	16.76889518	up
M001T2709	carboxyethyl-hydroxychroman	1.413615	0.003507	277384.5	up
M001T2716	1-(2,4,6-trihydroxyphenyl)ethan-1-one	1.398999	0.005171	110206.5	up
M001T2723	5-methyl-1,5-dihydrophenazin-1-one	1.454529	0.00078	3.769036329	up
M001T2735	1-(2-(p-Chlorophenoxy)-2-methylpropionyl)-3-(morpholinomethyl)urea	1.404345	0.004258	25.45569926	up
M001T2756	3-Indolehydracrylic acid	1.411683	0.003391	3.295251529	up
M001T2768	Glu Ala Asp	1.460507	0.000363	3.385293737	up
M001T2846	Dehydrocyanaropicrin	1.416794	0.002936	1892029	up
M001T2885	Methylhydroquinone	1.4334	0.001793	77.74140153	up
M001T2888	N,N-Dicyclohexylthiourea	1.430109	0.002126	9.965270476	up
M001T2917	2,4-dihydroxy-3,6-dimethylbenzoic acid	1.480207	2.87E-05	15.56115772	up
M001T2926	4-Hydroxy-3-(2-hydroxyethyl)acetophenone 4-glucoside	1.432741	0.002082	3.910726635	up
M001T2963	Isoscoparin 2-O-glucoside	1.410795	0.004168	74.73176083	up
M001T2976	2,6-Di-tert-butyl-1,4-benzenediol	1.474595	9.73E-05	15.54330099	up
M001T2985	2-(5-methoxy-2-methyl-1H-indol-3-yl)acetic acid	1.452179	0.000811	1493.260474	up
M001T2987	4-hydroxy-8-methoxyquinoline-2-carboxylic acid	1.470865	0.000129	5.03342975	up
M001T3014	(5alpha,6beta,14alpha,20R,22R)-5,6,14,20,27-Pentahydroxy-1-oxowith-	1.417478	0.003267	5.702292654	up

	24-enolide				
M001T3029	decyl acetate	1.470063	0.000185	13.45952818	up
M001T3069	Schidigeragenin C	1.410513	0.003446	6.486709305	up
M001T3079	Lariciresinol-sesquilignan	1.433891	0.001807	1961243	up
M001T3085	5-(2-Furanyl)-1,2,3,4,5,6-hexahydro-7H-cyclopenta[b]pyridin-7-one	1.404489	0.00472	20.51401009	up
M001T3100	Chenodeoxycholylaspartic acid	1.445125	0.001153	7.094545248	up
M001T3116	3,5-Dimethoxy-4-hydroxybenzaldehyde	1.434874	0.00188	3.210008959	up
M001T3124	1,3-Benzothiazol-2-amine	1.42039	0.00289	10.35591743	up
M001T3162	3-Methylthiopropyl-desulfoglucosinolate	1.453192	0.000638	12.13533513	up
M001T3166	2-(20-Hydroxyicosa-5,14-dienoylamino)acetic acid	1.403589	0.004272	5.509190667	up
M001T3181	dihydroartemisinic acid hydroperoxide	1.429671	0.002159	9.93755758	up
M001T3197	21-Deoxycortisol	1.400501	0.004966	6.602092838	up
M001T3223	(2S)-2-amino-3-({[(2R)-3-(hexadecanoyloxy)-2-hydroxypropoxy](hydroxy)phosphoryl}oxy)propanoic acid	1.417048	0.003112	17.97013037	up
M001T3231	Phosphocreatine	1.454312	0.000707	5.641618841	up
M001T3282	1,3-bis(methoxymethyl)-5,5-diphenyl-1,3-diazinane-2,4,6-trione	1.415119	0.003098	3.633375842	up
M001T3304	4-Methylcatechol	1.465408	0.000272	6.821042826	up
M001T3387	(3S)-1,4-epi-3-hydroxyacorenone	1.414998	0.003216	24.93875838	up
M001T3388	(3Z,6Z)-3,6-Nonadienal	1.422213	0.002332	3.304183641	up
M001T3390	(4-tert-butylphenyl)-[4-(4-methyl-6-pyrrolidin-1-ylpyrimidin-2-yl)piperazin-1-yl]methanone	1.407853	0.004256	55.59871208	up
M001T3555	3alpha-Hydroxy-11beta,13-dihydrodeoxymikanolide	1.447469	0.00084	16.05431499	up
M001T3570	4-Hydroxy-5-(dihydroxyphenyl)-valeric acid-O-methyl-O-sulphate	1.47496	6.52E-05	253.8734985	up
M001T3585	5-Butyl-3,4-dimethyl-2-furannonanoic acid	1.449205	0.000788	3.136875837	up
M001T3598	6-[(3-carboxy-3-methylprop-2-en-1-yl)oxy]-3,4,5-trihydroxyoxane-2-carboxylic acid	1.408478	0.003576	6.466820709	up

M001T3608	6-Peroxy-7(9)-dehydro-6,7-dihydrogeranyl acetate	1.395614	0.005441	5.632686332	up
M001T3609	6,10-Dimethylspiro[4.5]-dec-6-en-2,8-dione	1.463538	0.000433	3.450550961	up
M001T3628	7,8,4-Trimethylisoscutellarein	1.421712	0.00303	1492249	up
M001T3630	7a-Epialexaflorine	1.428808	0.0026	8.22903781	up
M001T3655	AN 11	1.435239	0.001779	499164.4	up
M001T3658	Annuionone F	1.478679	3.13E-05	72645	up
M001T3670	Asperorydine K	1.480259	1.93E-05	53.7277976	up
M001T3672	Atractyligenin (2-O-beta-glucopyranosyl-)	1.441423	0.001238	6.178852954	up
M001T3674	Avenasterol	1.403055	0.004755	29.04148415	up
M001T3677	Beclomethasone 17-monopropionate	1.423169	0.002368	5.489722483	up
M001T3700	Chaetomugilin Q	1.403546	0.004827	4.536963536	up
M001T3725	Columbaridione	1.447246	0.000857	15.19518408	up
M001T3739	Deacylcylindrol	1.412869	0.003757	4.087288304	up
M001T3741	Decarestrictin N	1.454904	0.000581	802625.2	up
M001T3742	Decarestrictine B	1.452784	0.000707	7.32049773	up
M001T3792	Flavone base + 3O, O-HexA-HexA	1.400142	0.004845	3.236191669	up
M001T3824	Halisulfate 8	1.448386	0.001058	914695.1	up
M001T3830	Hesseltin A photoisomer C	1.443565	0.001117	66.15655709	up
M001T3840	Hydroxytyrosol 4-glucuronide	1.468816	0.000188	4.941699679	up
M001T3845	Irisflavone C	1.437868	0.001461	185100.1	up
M001T3865	L-Tyrosyl-tRNA(Tyr)	1.430839	0.002176	123999.5	up
M001T3873	Lipoamide A	1.445095	0.000963	9.283584107	up
M001T3874	LPA(0:0/18:2(9Z,12Z))	1.406394	0.004386	6.68723062	up
M001T3909	MFCD00209793	1.452866	0.000615	6.020771139	up
M001T3915	Monnieraside I	1.450894	0.000728	1254640	up
M001T3927	N-[(2-fluorophenyl)methyl]-4-[(3-fluorophenyl)methyl]-3,5-dihydro-2H-	1.393601	0.005011	4.386778512	up

	1,4-benzoxazepine-7-carboxamide				
M001T3961	N-phenyl-3-[1-[6-(propylamino)pyrimidin-4-yl]piperidin-3-yl]propanamide	1.393438	0.005113	5.973982748	up
M001T3983	Nonadeca-10(Z)-enoic acid	1.461327	0.000443	33.51443524	up
M001T4044	Prostaglandin-c2	1.429046	0.001942	8.600998952	up
M001T4055	Rhodioloside E	1.472724	0.000157	8.232761965	up
M001T4106	Tschimganical A	1.468196	0.000217	325811.2	up
M001T4112	Urechitol B	1.485529	1.16E-07	474576.1	up
M001T4115	Verlotorin	1.435693	0.001878	39488.45	up
M001T4119	Withangulatin A	1.465225	0.000234	3.000939141	up
M001T4127	Zidovudine glucuronide	1.422116	0.00275	12.21952917	up
M001T0026	N-(1-Deoxy-1-fructosyl)phenylalanine	1.430733	0.00218	0.323733991	down
M001T0027	2-O-(alpha-D-Glucopyranosyl)glycerol	1.455279	0.000784	0.062649489	down
M001T0036	Jubanine A	1.485089	9.63E-07	0.014989061	down
M001T0055	Hebevinoside XIV	1.421543	0.002918	0.008377603	down
M001T0061	Phloretin-2-O-(2-O-xylosylglucoside)	1.406086	0.004596	0.048499758	down
M001T0093	STREPTOSETIN A	1.458336	0.000496	0.005524936	down
M001T0221	Phomalide	1.413827	0.003318	0.060769214	down
M001T0272	Asn Phe	1.465562	0.00029	0.173487788	down
M001T0274	Enterolactone	1.452051	0.000962	0.040348845	down
M001T0404	Oryzanol A	1.483552	6.03E-06	0.038476963	down
M001T0434	Val-glu-pro-ile-pro-tyr	1.414443	0.003206	7.22E-08	down
M001T0474	alanine lactate	1.475551	9.87E-05	0.009668359	down
M001T0515	1-Hexanol arabinosylglucoside	1.40455	0.004996	0.076520991	down
M001T0533	Cinncassiol D2 glucoside	1.417677	0.003486	0.237542408	down
M001T0543	Methyl 3b,24-dihydroxy-11,13(18)-oleanadien-30-oate	1.430178	0.001945	0.162213795	down

M001T0595	Goshonoside F6	1.433402	0.001651	0.029355737	down
M001T0596	Gibberellin A4 glucosyl ester	1.479262	3.75E-05	0.010939443	down
M001T0602	2,9-Dimethyl-2,9-diazatricyclo[10.2.2.25,8]octadeca-5,7,12,14,15,17-hexaene-3,10-diol, 9CI	1.458313	0.000563	0.113213518	down
M001T0616	Argenteane	1.464813	0.000247	1.80E-06	down
M001T0622	5-Hydroxy-6-methoxycoumarin 7-glucoside	1.482592	8.27E-06	0.274630374	down
M001T0632	23-trans-p-Coumaroyloxytormentic acid	1.480066	1.59E-05	1.91E-07	down
M001T0648	3-Methyl-2-butenyl 6-O-alpha-L-arabinopyranosyl-beta-D-glucopyranoside	1.468945	0.000177	0.200576233	down
M001T0734	[N,N-di-n-hexyl-2-(4-fluorophenyl) indole-3-acetamide]	1.466876	0.000256	0.014833632	down
M001T0833	Serylleucine	1.454451	0.000801	0.20356078	down
M001T0847	(2R)-2-hydroxy-6-{[(4-hydroxy-2-methoxyphenyl)methyl]imino}-2,5-dimethylcyclohex-4-ene-1,3-dione	1.414489	0.00397	0.31920476	down
M001T0877	Ala Ala Ile	1.447943	0.001059	0.078585235	down
M001T0939	Taurocholic acid 3-sulfate	1.480735	2.84E-05	0.00135105	down
M001T0965	Gln Gln Leu	1.407765	0.004681	0.025900067	down
M001T0966	Gln Glu Ile	1.474177	0.000106	0.208717458	down
M001T0977	Asn Ile	1.46263	0.000463	0.190028495	down
M001T0982	Ser Lys	1.457174	0.000478	0.122831213	down
M001T1028	(2S)-2-[(2S)-2-amino-4-carbamoylbutanamido]-3-carbamoylpropanoic acid	1.455548	0.000589	0.187470894	down
M001T1066	Valinopine	1.474575	8.70E-05	7.80E-07	down
M001T1083	3-Palmitoylcatechin	1.480652	2.58E-05	0.000574347	down
M001T1089	Ganoderiol C	1.405906	0.004606	0.330702424	down
M001T1093	Manassantin A	1.447565	0.00099	0.086956462	down
M001T1113	(2S,4S,6S)-6-[2-(carboxyamino)phenoxy]-3,4,5-trihydroxyoxane-2-	1.437333	0.001868	0.008050143	down

	carboxylic acid				
M001T1144	glucose lactate ketone	1.414498	0.003022	1.98E-05	down
M001T1155	MG(0:0/20:3(6,8,11)-OH(5)/0:0)	1.437587	0.001533	0.193547595	down
M001T1164	PA(18:3(9,11,15)-OH(13)/8:0)	1.464823	0.000363	8.38E-05	down
M001T1171	PG(PGD1/20:3(8Z,11Z,14Z))	1.440035	0.001507	0.011677147	down
M001T1199	DG(18:3(9,11,15)-OH(13)/0:0/10:0)	1.428129	0.001914	0.11921086	down
M001T1230	Evobioside	1.453858	0.00059	0.027145893	down
M001T1257	Cortolone 3-glucuronide	1.425775	0.002861	0.107024528	down
M001T1285	N6-Carbamoyl-L-threonyladenosine	1.465212	0.000347	0.004475326	down
M001T1306	Pneumadin	1.481075	2.91E-05	0.002020701	down
M001T1368	Asn Asn Leu	1.479421	2.47E-05	0.022416899	down
M001T1371	Threoninyl-Isoleucine	1.476234	6.44E-05	0.218590275	down
M001T1378	4-(Methylnitrosamino)-1-(3-pyridyl)-1-butanol glucuronide	1.426553	0.002517	0.222577825	down
M001T1415	D-Lysopine	1.437157	0.00194	0.196723685	down
M001T1459	Dihydrodemethylsterigmatocystin	1.418807	0.002966	0.059534046	down
M001T1476	(-)-5-oxo-1,2-campholide	1.468186	0.000294	0.134384944	down
M001T1498	Cnidioside A	1.468007	0.000261	0.167692079	down
M001T1499	Mudanoside B	1.48041	2.73E-05	3.48E-06	down
M001T1501	Madlongiside C	1.480164	2.78E-05	0.020948208	down
M001T1530	.+/-.-2-Amino-1-butanol	1.445352	0.000937	0.235571064	down
M001T1570	Daidzein 7-glucuronide-4-sulfate	1.455627	0.000693	0.018405031	down
M001T1576	N-Hydroxy-L-tryptophan	1.454596	0.000639	0.06824413	down
M001T1619	2-[(2E)-3-(3,4-dihydroxyphenyl)prop-2-enamido]butanedioic acid	1.442179	0.001309	0.010441803	down
M001T1628	Bacillaene	1.473086	8.19E-05	0.014644579	down
M001T1653	2-(2-amino-4-methylpentanamido)propanoic acid	1.428641	0.002482	0.135660722	down
M001T1727	Lucyoside R	1.425182	0.002581	0.070051857	down

M001T1761	2-{1,8-diethyl-1H,3H,4H,9H-pyrano[3,4-b]indol-1-yl}acetic acid	1.475834	9.89E-05	0.251474575	down
M001T1813	2-Hydroxyethanesulfonate	1.461824	0.000401	0.079333856	down
M001T1818	Histidinyl-Isoleucine	1.431264	0.001675	0.114823167	down
M001T1825	Trans-3-Hydroxycotinine O glucoronide	1.41831	0.003533	0.252394182	down
M001T1868	(2S,4S)-pentane-1,2,3,4,5-pentol	1.447324	0.001218	0.168558377	down
M001T1917	(2R)-2-beta-D-Glucopyranosyloxy-4-hydroxy-7-methoxy-(2H)-1,4-benzoxazin-3(4H)-one	1.438787	0.001504	0.017320329	down
M001T1945	Swertiamarin	1.47797	6.96E-05	0.062524397	down
M001T1948	Medicarpin 3-O-(6-malonylgluclside)	1.405369	0.004542	0.004379217	down
M001T1956	Quercetin 7-rutinoside	1.425636	0.002727	4.81E-07	down
M001T1965	Glycochenodeoxycholic acid 3-glucuronide	1.483417	2.97E-06	0.03530645	down
M001T1971	7-Hydroxymitragynine	1.449539	0.000948	0.264716042	down
M001T1979	Cyclo(D-trp-D-asp-pro-D-val-leu)	1.412323	0.00427	0.298363782	down
M001T1997	(2E)-3-phenylprop-2-enoic acid	1.436046	0.001585	0.118291828	down
M001T2011	L-Fucitol	1.425683	0.002337	0.123730015	down
M001T2034	Isoleucyl-Valine	1.465307	0.000386	0.045719178	down
M001T2062	O-Oxalylhomoserine	1.474702	0.00012	0.041423273	down
M001T2084	2-amino-2,3,7-trideoxy-D-lyxo-hept-6-ulosonic acid	1.442091	0.001087	0.11216053	down
M001T2108	7-[(2,6-dihydroxy-2,5,5,8a-tetramethyl-decahydronaphthalen-1-yl)methoxy]-2H-chromen-2-one	1.407014	0.004624	0.096008195	down
M001T2115	(2S,3S,4S,5R,6S)-6-[5-(2-carboxyethyl)-2-methoxyphenoxy]-3,4,5-trihydroxyoxane-2-carboxylic acid	1.459265	0.000456	0.009071095	down
M001T2155	5,7-dihydroxy-2-(4-methoxyphenyl)-4H-chromen-4-one	1.411327	0.003945	0.16183237	down
M001T2156	5,7-dihydroxy-2-(4-hydroxyphenyl)-4H-chromen-4-one	1.417945	0.002984	0.231386289	down
M001T2186	Cucurbitacin A	1.444118	0.001145	0.014675896	down
M001T2358	LysoPE(0:0/22:1(13Z))	1.470025	0.000207	0.002833167	down

M001T2439	6-Dehydrotestosterone glucuronide	1.44617	0.001254	0.120590494	down
M001T2470	Phe Ala	1.479326	4.49E-05	0.064791639	down
M001T2493	Saccharumoside C	1.436547	0.001767	0.003786638	down
M001T2529	Cycloartanyl ferulate	1.408269	0.004628	0.056350086	down
M001T2536	(2S)-2,6-diaminohexanoic acid	1.427024	0.002201	0.302349167	down
M001T2560	(2S)-2-amino-3-phenylpropanoic acid	1.422284	0.002597	0.14089287	down
M001T2582	(2S,3S)-2-amino-3-methylpentanoic acid	1.47469	6.50E-05	0.127914914	down
M001T2585	Val Leu Ser Pro Ala	1.434188	0.001899	2.81E-07	down
M001T2610	Fusarin C	1.478794	2.28E-05	0.042065252	down
M001T2643	(5E)-N-[(4-hydroxy-3-methoxyphenyl)methyl]-7-methyloct-5-enamide	1.46346	0.000295	0.227281246	down
M001T2724	Flindersine	1.442903	0.001276	0.196297842	down
M001T2765	2-[(2S)-2-amino-3-phenylpropanamido]acetic acid	1.413541	0.003938	0.225134174	down
M001T2771	H-LEU-VAL-OH	1.470516	0.000221	0.146923479	down
M001T2778	(2S)-2-[(2S)-2-amino-3-hydroxypropanamido]-3-phenylpropanoic acid	1.416723	0.003462	0.122009942	down
M001T2791	Alanyl-Lysine	1.481264	1.27E-05	0.047335676	down
M001T2802	GLY-GLY-ILE	1.449076	0.001075	0.133542661	down
M001T2805	Ile Arg	1.423221	0.002274	0.074486883	down
M001T2807	(2S)-2-[(2S,3R)-2-amino-3-hydroxybutanamido]-4-methylpentanoic acid	1.47467	5.95E-05	0.009643899	down
M001T2828	(2R)-2,5-diaminopentanoic acid	1.456359	0.000559	0.025038106	down
M001T2848	(2R,3R)-3-Methylglutamyl-5-semialdehyde-N6-lysine	1.483821	4.74E-06	0.009273564	down
M001T2856	Leu Thr Ala	1.468378	0.000272	0.070541714	down
M001T2902	2-[2-hydroxy-3-(3-hydroxy-4,5-dimethoxyphenyl)propoxy]-6-(hydroxymethyl)oxane-3,4,5-triol	1.463736	0.000397	0.121731202	down
M001T2915	Tetrahydro-2-furanmethanol	1.432563	0.002056	0.077886767	down
M001T2934	Lansioside C	1.466721	0.000299	5.83E-07	down
M001T3013	Ambonic acid	1.428916	0.002107	0.041543874	down

M001T3027	Piperidine	1.441257	0.001357	0.161695329	down
M001T3030	(2S)-2-[(2S)-2-amino-4-methylpentanamido]propanoic acid	1.434847	0.002005	0.102293047	down
M001T3039	Paracetamol sulfate	1.40074	0.004254	0.311866298	down
M001T3072	Lucuminic acid	1.471364	0.000175	0.006843517	down
M001T3087	Citrusin I	1.467389	0.000194	0.001531571	down
M001T3114	Gentamicin A	1.419935	0.002706	0.27063138	down
M001T3140	Gly-Ile	1.457475	0.000657	0.314675892	down
M001T3205	Gly-L-Phe	1.458454	0.000479	0.261393754	down
M001T3237	Ceftizoxime alapivoxil	1.404345	0.004591	0.078311561	down
M001T3240	(2S)-2-[(2S)-2-aminopropanamido]-3-phenylpropanoic acid	1.4811	2.48E-05	0.203305698	down
M001T3242	Trp Gly	1.440538	0.001252	0.319280744	down
M001T3346	(+)-Setoclavine	1.47815	3.56E-05	0.033490977	down
M001T3379	(2S,3R)-3-(4-Hydroxyphenyl)-2-(4-{[(2S)-2-pyrrolidin-1-ylpropyl]oxy}phenyl)-2,3-dihydro-1,4-benzoxathiin-6-OL	1.409801	0.004113	0.18969472	down
M001T3460	13:4+4O fatty acyl hexoside	1.47204	0.000117	0.040288344	down
M001T3482	2-[3-oxo-6-(piperidine-1-carbonyl)-1,4-benzothiazin-4-yl]-N-(3-piperidin-1-ylpropyl)acetamide	1.405632	0.003848	0.146808402	down
M001T3492	2-Hydroxyethyl gardenamide A	1.407717	0.004324	7.71E-06	down
M001T3597	6-(3-methylphenyl)-7-[2-oxo-2-(4-pyridin-2-ylpiperazin-1-yl)ethyl]-7H-pyrrolo[3,4-b]pyridin-5-one	1.423875	0.002948	0.177303195	down
M001T3618	6e,9e-Dihydroxy-4,7E-megastigmadien-3-one 9-[apiosyl-(1->6)-glucoside]	1.45326	0.000873	0.001567514	down
M001T3620	7-(3,4-dimethoxyphenyl)-2-(4-pyridin-2-ylpiperazin-1-yl)-7,8-dihydro-6H-quinazolin-5-one	1.444946	0.001231	0.140849527	down
M001T3708	CHEBI:73506	1.448791	0.000903	0.175920052	down
M001T3717	CI-959	1.477562	7.05E-05	0.046663127	down

M001T3729	Cotinineglucuronide	1.442554	0.001193	0.025111315	down
M001T3754	Di-demethylsimmondsin	1.46928	0.000188	0.041608947	down
M001T3764	DIMBOA + O-Hex	1.475199	7.27E-05	0.001549976	down
M001T3771	Ebracteatoside A	1.443588	0.001344	1.17E-07	down
M001T3778	Ergosecaline	1.421944	0.003036	0.075993983	down
M001T3785	FA 18:2+2O	1.439947	0.001588	0.058876976	down
M001T3815	Gonyautoxin IV	1.415388	0.003079	0.034339891	down
M001T3822	H-Ile-Gln-OH	1.454498	0.000858	0.126682192	down
M001T3823	H-Ser-Leu-Ile-Gly-Arg-Leu-OH	1.394697	0.004849	0.044283081	down
M001T3871	Licodione base + 3O, 2Prenyl	1.443873	0.001158	0.161321174	down
M001T3914	Milbemycin beta2	1.408089	0.003554	0.147141871	down
M001T3922	N-(2,4-difluorophenyl)-4-(2-oxo-2-piperidin-1-ylethyl)piperidine-1-carboxamide	1.437506	0.00161	0.252675775	down
M001T3980	Nitrobacter ferritin	1.407569	0.003667	4.99E-07	down
M001T4009	PE(PGF1alpha/P-16:0)	1.478857	4.65E-05	1.17E-06	down
M001T4030	PI(LTE4/18:0)	1.429144	0.001824	0.015636092	down
M001T4086	Terreustoxin K	1.425985	0.002063	0.036534511	down
M001T4104	Trigilletimine	1.459115	0.000487	0.004574448	down
